# The Effects of Biogeography on Ant Diversity and Activity on the Boston Harbor Islands, Massachusetts, U.S.A

**DOI:** 10.1371/journal.pone.0028045

**Published:** 2011-11-29

**Authors:** Adam T. Clark, Jessica J. Rykken, Brian D. Farrell

**Affiliations:** 1 Harvard College, Cambridge, Massachusetts, United States of America; 2 Museum of Comparative Zoology, Harvard University, Cambridge, Massachusetts, United States of America; 3 Department of Organismic and Evolutionary Biology, Harvard University, Cambridge, Massachusetts, United States of America; Monash University, Australia

## Abstract

Many studies have examined how island biogeography affects diversity on the scale of island systems. In this study, we address how diversity varies over very short periods of time on individual islands. To do this, we compile an inventory of the ants living in the Boston Harbor Islands National Recreation Area, Boston, Massachusetts, USA using data from a five-year All Taxa Biodiversity Inventory of the region's arthropods. Consistent with the classical theory of island biogeography, species richness increased with island size, decreased with island isolation, and remained relatively constant over time. Additionally, our inventory finds that almost half of the known Massachusetts ant fauna can be collected in the BHI, and identifies four new species records for Massachusetts, including one new to the United States, *Myrmica scabrinodis*. We find that the number of species actually active on islands depended greatly on the timescale under consideration. The species that could be detected during any given week of sampling could by no means account for total island species richness, even when correcting for sampling effort. Though we consistently collected the same *number* of species over any given week of sampling, the *identities* of those species varied greatly between weeks. This variation does not result from local immigration and extinction of species, nor from seasonally-driven changes in the abundance of individual species, but rather from weekly changes in the distribution and activity of foraging ants. This variation can be upwards of 50% of ant species per week. This suggests that numerous ant species on the BHI share the same physical space at different times. This temporal partitioning could well explain such unexpectedly high ant diversity in an isolated, urban site.

## Introduction

MacArthur and Wilson's classical theory of island biogeography posits that diversity on islands is governed by the rates of local immigration and extinction of species from the mainland, and is thought to be the dominating process determining species richness on islands [1]–[5]. As a result the theory suggests that though species composition on an island may vary across time, the actual number of species on that island remains constant [2], [6]. That is, there is some equilibrium species number for each island depending on the island's physical characteristics. In particular, the classical theory of island biogeography identifies relationships between the number of species found on an island, the island's area, and its isolation from the mainland: Large islands and islands close to the mainland are expected to have comparatively more species than small or isolated islands [3], [7]–[11].

Just as species richness varies between islands, there are likewise differences in the abundance and activity patterns of species across a single island. It is relatively intuitive that across space, and particularly across different habitats, species composition changes. After all, with the notable exception of human beings [12], no single species has ever come to dominate the entire biosphere [13]. Living organisms seem to face some sort of an ecological tradeoff in which success and specialization in a particular area necessarily comes at a cost to other traits [14], [15].

The degree to which these tradeoffs govern even the small-scale interactions between species is controversial [16]. Classical niche theory suggests that small differences in species' resource requirements ultimately determine the circumstances under which they can coexist [17]–[19]. By definition, a particular assemblage of species can only coexist given that no one species' use of resources precludes the minimum requirements of another: if two co-occurring species are too ecologically similar, one of them is bound to die out eventually [20]. Only when resource tradeoffs prevent a single species from depleting resources below the thresholds required by other species in the assemblage is coexistence possible [19].

A variation on this question that is not explicitly addressed by the classical theory of island biogeography is when and how species are able to coexist by sharing the same *space* at different *times*. In a heterogeneous ecosystem filled with many different species assemblages, there can be a great deal of flow of species between habitat patches. Though it is theoretically understood that movement between patches and temporal variation in foraging activity can encourage coexistence of otherwise mutually exclusive species [21]–[23], little empirical work has addressed this [24], particularly in small arthropod ecosystems.

Here, we examine the biogeography and community ecology of ants in the Boston Harbor Islands National Recreation Area in Boston, Massachusetts (BHI). Our study is motivated by the simple observation that 51 ant species coexist in the BHI, a small island park system located just outside of downtown Boston. We ask how so many ecologically similar species can coexist in such an isolated and disturbed natural environment. We argue that this is accomplished by temporal partitioning of niche space. To do this, we focus not on the large scales commonly presented in studies of island biogeography, but rather on small time periods in a high-resolution study of the region's fauna.

High ant diversity in the BHI is surprising for a number of reasons. The entirety of Massachusetts is known to harbor around one hundred ant species (S. Cover, personal communication). However, the BHI represents a much smaller area and fewer habitats (see Supporting Information S5). Additionally, islands are expected to be species depauperate in comparison to an equivalent area in mainland systems [2]. Moreover, the BHI have a long history of human-induced disturbance and changes to land cover (See Supporting Information S4). In studies where ant populations were subjected to disturbance, and particularly habitat fragmentation, a significant decrease in ant species richness was observed [25], [26]. These decreases in species richness were accompanied by the replacement of native species by exotic and “tramp” ant species [27]. On all counts, ant communities in the BHI should be relatively homogenous and composed of comparatively few species in relation to mainland Massachusetts.

We base our study on a five-year All Taxa Biodiversity Inventory (ATBI) of the arthropods of the BHI. The goals of our study were (1) compile an inventory of the ants of the BHI based on the ATBI; (2) determine how these species are distributed across space and time; and (3) assess how these species are able to coexist in a spatially bounded ecosystem. Using these data, we then crafted a model showing how variations through time in the ant species assemblages present in our sampling plots could explain observed changes in ant abundance. Based on the classical theory of island biogeography, we expected that the number of ant species would differ among islands but be constant through time on any particular island, reflecting a stable equilibrium. However, we hypothesized that the total number of ant species on any given island would be significantly larger than the number of species actually contemporaneously co-occurring because of temporal partitioning of resources. That is, that many ant species would end up sharing the same physical space on islands at different times.

## Methods

### Inventory

**Figure 1:**
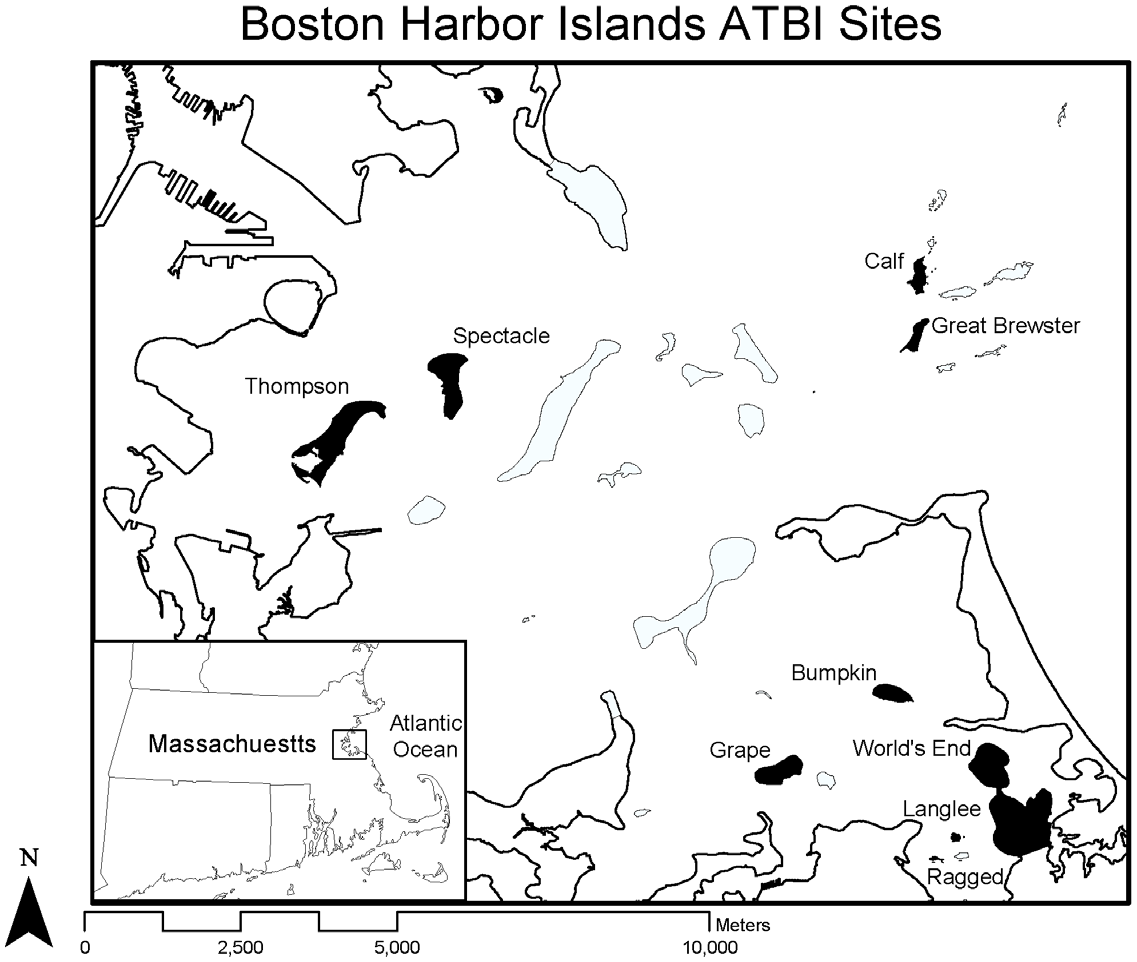
The Boston Harbor Islands National Recreation Area (BHI). Islands in the park are colored gray. Islands that were intensely sampled as part of the ATBI during the summers of 2005–2009 are colored black. Map shapefiles from MassGIS and NOAA [28], [61].

The BHI is a collection of 34 islands and peninsulas outside of downtown Boston, Massachusetts, USA (Figure 1). The park represents a myriad of historical land uses, ranging from ancient American Indian settlements to pasture, military prisons, and garbage dumps. The islands range in size from about one to one hundred hectares, and are separated from the mainland by a few dozen meters to over six kilometers [28]. Although the range and maxima of island sizes and distances from the mainland are small relative to many island biogeographic studies, they provide several orders of magnitude of variation and there is already strong evidence that the rules of biogeography apply to the BHI and similar island systems at these scales [9], [27].

Since 2005, the ant diversity of ten islands in the BHI has been sampled as part of an ATBI of the region's invertebrates. Ants are an excellent model taxon for this study because they are ecologically diverse and abundant in most terrestrial ecosystems [29], including all of our study sites. Additionally, ants make up an appreciable fraction of animal biomass and are dominant components of invertebrate communities [29], [30], even in New England. Finally, ant communities are highly and predictably structured [31]–[33], and together with plant community composition, have been suggested as a tool for informing management plans [34]–[36].

The natural history of ants suggests that temporal partitioning of resources could be important to the coexistence of species. All ants are eusocial, and the basic unit of ant life is the colony [37]. A “dispersal” event for ants is therefore not constituted by the movement of individual workers, but rather of a fertilized queen or a nest. As such, ant dispersal and particularly the founding of new colonies on islands is extremely limited because virgin queens only mate and fly to new nest sites during a few crucial weeks of the year [38]. This means that dynamics in species communities over the course of a single year cannot be explained by the standard immigration-extinction patterns seen in more vagile species.

We sampled 10 islands in the BHI, varying in island size and distance from mainland, from early May through late October during each of the summers of 2005 through 2009. We selected between 10 and 30 sites on each island, depending on island size. Sites were selected non-randomly in order to include as many habitat types as possible (Table 1). We used a variety of collection methods including: baiting, bee-bowls, net- and hand-collecting, litter-sifting, malaise traps, mercury-vapor and ultra-violet light traps, and pitfall traps. Some of these methods are not commonly used for collecting ants, but are standard procedures for comprehensive collection of arthropods as part of an ATBI. Methods were standardized among islands, and all islands contained a diverse mixture of sampling procedures. For the BHI, we obtained permits from the NPS, permit number BOHA-2006-SCI-0004, to collect terrestrial arthropods, and received permission from islands' individual owners (Massachusetts Department of Conservation and Recreation, Thompson Island Outward Bound, Town of Hingham, and the Town of Winthrop) to collect on their land.

**Table 1:** Sampling statistics for ants in the BHI from the 2005–2009 ATBI, area and isolation data from MassGIS [28].

Island	area (km^2^)	isolation (km)	samples	*S_obs_*	abundance	vegetation class (Elliman, 2005)
**Bumpkin**	0.12	0.64	53	27	885	woodland, shrub, field
**Calf**	0.07	3.29	117	22	972	shrub, salt marsh
**Grape**	0.22	0.47	179	37	4506	woodland, shrub, field
**Gr. Brewster**	0.08	2.36	130	22	1481	shrub, salt marsh, field
**Langlee**	0.02	0.52	80	32	977	woodland, shrub
**Ragged**	0.01	0.32	97	27	1378	woodland, shrub
**Snake**	0.03	0.35	50	22	752	salt marsh
**Spectacle**	0.35	1.92	138	22	1251	shrub, field
**Thompson**	0.54	0.51	383	40	3993	woodland, shrub, salt marsh, field
**Word's End**	1.08	0.00	193	40	1366	woodland, shrub, shrub swamp, field

*Notes:* Abbreviations are as follows: Area indicates terrestrial area above high tide line. Isolation indicates distance between island and nearest mainland. Samples indicates the number of sampling events that took place on each island. *S_obs_* indicates the total number of ant species collected. Abundance indicates the number of ant individuals collected. Area and isolation data from MassGIS [28].

To collect data on the abundance, location, and phenology of arthropods on the BHI, we set up pitfall and malaise trap sites on the islands every two weeks. Traps were left open to collect specimens for a week, and then closed for a week to avoid harmful population reductions. We changed the location of malaise sites every two weeks, whereas we monitored permanent pitfall trap sites for the entire sampling season. Additionally, we used several short-term sampling methods. On each island, we conducted at least one overnight sampling using mercury-vapor and ultra-violet light traps. We also employed a variety of hand-collecting techniques, including hand-sampling from vegetation, leaf-litter sifting, beat-sheets, and aerial- and beat-nets. Large “BioBlitzes” were also organized on several islands, where large groups of volunteers joined us on for a day of intensive hand-sampling. We stored specimens in 95% ethanol in the Museum of Comparative Zoology (MCZ) at Harvard University, Cambridge, MA, where we identified the ants to species. We pinned voucher specimens from each collecting event, and returned the remaining specimens to 95% ethanol for long-term storage. All specimens are deposited at the MCZ.

To account for the differences in sampling regimes, we constructed rarefaction curves and compared species detection among islands and sampling methods. Rarefaction curves estimate the number of species that we expect to collect given increasingly large samples, based on randomized re-sampling from the total pool of collecting events [39], [40]. These simulations are repeated many times, typically at least 1,000, and the average number of species expected from a particular sampling effort is then calculated to assess relative species richness [39]. Rarefaction curves are a common method for standardizing comparisons of species richness between samples of differing sizes. This is important, because it helps determine whether differences in the number of species collected on different islands are the result of actual differences in species richness, or are the result of sampling bias.

We conducted our simulations in R [41] by randomly sampling species from the total pool of sampling events. As species from each new collecting event were added to the curve, we recorded the number of species and individuals expected from the corresponding sampling effort. For our analyses, it is important to note that we considered ”species occurrences” – that is, the number of times each species was collected in independent sampling events – rather than the occurrence of individual ants. This is to account for ants' nest-centered ecologies: High abundance in a sample for a particular species reflects both the proximity and size of its nest as much as it reflects high abundance in the landscape at large [29].

Based on 1,000 iterations, we plotted the average number of species collected corresponding to the abundance of species collection events, and computed a 95% confidence interval based on our simulations. This interval represents a null model for our analysis, showing the number of species we expect to collect from a particular sampling effort in our study, given no significant bias in sampling methods [39]. We also compared our rarefaction curves to the MaoTau sample-based rarefaction estimate from the popular ecological statistics program EstimateS version 8.2.0 (See Supporting Information S2).

### Estimate of active species

To estimate the number of species actually coexisting in space and time, we need a way to measure the number of species that are actually active in our sample sites. Any spatially bounded region harbors a finite number species at any given time. Because of this, rarefaction curves tend towards an asymptote as sampling effort approaches infinity. This is in contrast to species-area curves, which sample increasingly large regions, and therefore tend towards infinite species diversity at very large spatial scales [2]. It should therefore be possible to fit an asymptotic function to a rarefaction curve and extrapolate an approximation of total regional diversity to account for the inevitability of incomplete sampling of rare species [39], [42].

Moreover, the asymptote of the curve, which shows the number of new species that could still be collected by increasing sampling effort, has been shown to provide accurate estimates for a region's total diversity even for very small sample sizes [43]. Such an asymptotic estimate derived from observed species occurrences estimates the total number of “active species” – that is, the number of species that could be collected given an infinite sampling effort over the spatial and temporal scales represented by the sampling. This method is analogous, but not equivalent, to species richness estimators such as Chao I and II [42], which estimate overall species richness based on the observed number of species. This is particularly important, because even long-term and intensive surveys are unlikely to discover all species present in a given area [39], [44].

For each of our 1,000 iterated rarefaction curves, we estimated the function's asymptote *sensu* Rosenzweig *et al.* (2003) by fitting a logistic curve of the form *S_obs_* = *S∧[(−N∧(−qN∧q)]* where *S_obs_* is the observed number of species collected given *N* sampling events, *S* is the rarefaction curve's asymptote, or the number of species expected from an arbitrarily large sampling effort, and *q* is a fitted constant. We then calculated the mean estimate for *S* and corresponding standard deviation from our simulations. While we are not aware of any biological significance of the function other than the shape of the resulting curve, it was chosen from a set of several asymptotic functions because it fit the data extremely well even for very small sample sizes [43].

We repeated this procedure for each island, building rarefaction curves from the pool of all individuals collected only on that island. Additionally, we estimated *S* for specific time slices on each island by separating our sample pools by week (i.e. individuals collected during the n^th^ week of the year on a particular island). We omitted weeks containing fewer than five sampling events to ensure sufficient data for the analysis. Nonetheless, because our sample sites were chosen to maximize the number of habitats sampled, even very small sample sizes should provide accurate rarefaction-based estimates of *S* [43].

Using the results from our asymptotic curve fitting, we estimated the expected number of active species on each island, both for the entire sampling season and an average week of sampling. For each simulated series of rarefaction curves, we calculated the mean asymptotic estimate of *S* and the corresponding variance. Because the curve fitting procedure used to estimate *S* occasionally fails or converges to unrealistic values, we first removed all estimates of “*S* = 0”, as well as the top and bottom 10% of asymptotic estimates. Because ants were detected every week on every island, *S* = 0 reflects model fitting error rather than zero ant activity. We then compared expected *S* for the sampling season against the average weekly *S* to determine how species composition changed on each island over time. Additionally, we compared our asymptotic estimates to the sample-based Chao II species estimate calculated in EstimateS (See Supporting Information S2).

### Determinants of species activity

To assess diversity patterns across islands, we calculated three statistics that address the “density” of species on an island, differences in diversity across space, and differences across time respectively. First, we recorded the mean number of species collected per sampling event on each of the islands. Second, the spatial turnover, or heterogeneity of species composition between sampling events as *spatial turnover = S/mean number of species per sampling event* [45]–[47], or the expected proportion of total island diversity that can be accounted for by sampling at a single site. Finally, we calculated weekly temporal turnover for the BHI and each island using Bray Curtis dissimilarity, which estimates the fraction of species not shared between two sequential sampling events in a given region. This index ranges from 0 (all species shared between sites) to 1 (no species shared between sites).

We then compared these quantities between islands to assess the effects of island isolation and area. We first used simple linear regression of our data to assess the significance and power of these relationships. Then, using island isolation from the mainland (in km from shore) and island terrestrial area (in km^2^ of land above the high tide mark), we grouped islands into two levels for each analysis: “near” islands (0–1.65 km) and “far” islands (1.65–3.29 km), “small” islands (0–0.54 km^2^) and “large” islands (0.54–1.08 km^2^). These cutoffs were chosen based on preliminary analysis of biogeographic patterns in order to increase the power of our tests, and put half of the islands into each category. Using a fixed-factor ANCOVA of island isolation and size against week of the year, we assessed whether estimated number of active species, mean species detected per sampling event, spatial turnover, or temporal turnover differed significantly between islands or between time periods.

Additionally, to account for potential seasonal changes in ant species composition, we assessed annual trends associated with ant diversity using both empirical orthogonal analysis [48] and the empirical Bayes approach for identifying non-random species associations [49] (See Supporting Information S3). Second, in order to account for differing levels of anthropogenic disturbance on islands, we compared the number of species detected among biogeographically similar islands with differing disturbance regimes, and between experimental plots before and after they were subjected to simulated disturbance. In these experimental plots, we removed all vegetation and ant nests present in 1-by-1-meter transects at the beginning of the sampling season and tracked corresponding changes in ant species abundance (See Supporting Information S4). To account for the possible confounding influences of Words End, which is actually a peninsula connected to the mainland by a narrow bottleneck, we also repeated all analyses with Worlds End removed (See Supporting Information S7).

### Community dynamics modeling

Finally, we hoped to identify the actual species behind changes in observed activity patterns. Identifying the constituent members of the ant communities themselves required special attention. Analysis of nonrandom species associations is inherently problematic because of the large number of possible comparisons. Looking at all possible pairs of species in a moderately sized dataset – say, fifty species – would result in 1,225 possible comparisons. If we also care about species assemblages of size three, this number rapidly grows to 20,825. To address all possible species assemblages of size *m* or smaller given *n* species, we would need make the binomial sum of *n* choose *m*, or ∑ *binomial*(*n,m*), comparisons. This leads to unacceptably large type I error. At any given statistical alpha (for example, the traditional alpha = 0.05) a large number of random species assemblages will appear to be to be significantly non-random simply due to chance, and it will be impossible to separate significant species pairs from statistical anomalies.

This problem can be addressed in several ways. One is to use a statistical correction, such as the Bonferroni correction. This technique reduces type-I error by lowering alpha, at the expense of statistical power. However, this technique risks mislabeling meaningful combinations of species as statistically insignificant – that is, it increases type-II error. An alternative approach, recently engineered by Gotelli and Ulrich, takes note of the expected number of co-occurrences based purely on species abundance, and uses this to tease out significantly correlated pairs of species [49]. Again, however, this technique risks under-representing significant relationships between species.

In our model, we reduce type I error by testing fewer combinations of species. Rather than trudging through every possible species combination in the search of ant communities, we focus on only those assemblages that actually occur on the BHI, and use them to construct likely communities capable of generating the patterns observed in our samples. We do this by identifying the observed frequency with which each species is seen to replace others in subsequent sampling events. We then transform these data on the progression of assemblages into stochastic Markov transition matrices for analysis. To account for missing records of species in our dataset, we used a simple capture-recapture technique [5]. In any instance where a species disappeared and reappeared at a particular plot between sampling events, we assumed its presence throughout the sampling period. Additionally, to facilitate analysis, we removed records of very rare species, retaining only the *n* most common species that accounted for 95% of collection events.

To identify significant transitioning communities, we enumerated all possible assemblages found in our sampling data. Using a G-test for independence [50], we combined any assemblages that were statistically indistinguishable (p≤0.05) from one another by taking their intersect – that is, we retained all species common to both assemblages in a new assemblage class. Based on these assemblages, we tabulated the total number of transitions between assemblage states that took place through time in our plots in a transition matrix, for example, the number of times that assemblage *A* in a plot changed to assemblage *B* between two sequential sampling events.

Next, using a modified version of Bossert's stochastic finite sequence generator algorithm [51], we simplified the transition matrix by combining assemblages with similar transition properties. This algorithm creates a series of “states” in a Markov transition matrix that can be used to produce a sequence statistically indistinguishable from the sequence being analyzed. For our purposes, it computes a list of potential transition probabilities that could explain species assemblage patterns observed on the BHI. In this algorithm, any assemblages with statistically indistinguishable columns in the transition matrix (using a G-test, p≤0.05) are combined, again by taking the intersect of the two assemblages. Based on these matrices, we characterized dominant assemblages using their Eigen values to construct a stable state distribution. We identified these dominant assemblages as significant “communities”. We converted the resulting reduced transition matrix into a stochastic transition matrix, and again calculated the stable state distribution of each assemblage, which approximates the relative length of time that each assemblage is expected to persist at sample sites.

Lastly, we repeated the entire community modeling exercise on subsets of the data to analyze how these dynamics were affected by changes in habitat type and disturbance regime, two factors that varied greatly between sites in the BHI. We repeated the analysis using the 1) entire ATBI dataset, 2) samples taken in open, shrubby and forested habits (average height of vegetation <0.1-meters, <2-meters, and >2-meters respectively, See Supporting Information S5 for habitat inventory information), and 3) data from a 2009 plot disturbance experiment (See Supporting Information S4 for methods). We then compared the predicted community structure and transition probabilities for each of these subsets.

A few caveats should be kept in mind regarding this method. First, because species communities are assembled using the intersect of community states (that is, AB+BC = B), the model measures only for the presence of particular species groups, not for absence. Additionally, this means that ecologically equivalent communities with interchangeable species, say ABC and BCD, will only include shared community members, BC, even if the additional species is important to community structure. Moreover, transitions are counted more than once per time step: AB→BC simultaneously includes the transitions A→B, B→B, A→BC, B→BC, etc. Transition probabilities still imply a direction to most relationships – that is, the relative number of times that a particular transition take place – but do not necessarily imply complete exclusion of particular species. Additionally, the stable state distribution cannot be interpreted as a probability vector, despite the fact that it is normalized to unity by convention. Though it gives information on the magnitude of time spent in each community state – that is, state A persists on average more often than state B does – the stable state distribution does not represent the probability of being in each state at any one moment in time, because the states are not mutually exclusive.

## Results

We hypothesized that the number of active species would differ between islands, but be constant through time. In total, we completed about 1,400 sampling events and collected almost 18,000 individual ants in about 3,400 species occurrences. From our collections, we identified 51 species, 20 genera, and 4 subfamilies (Tables 2, 3, see Supporting Information S1, S8 for species checklist and collection data). Among these species were *Anergates atratulus*, *Pyramica metazytes*, and *Camponotus caryae*, three new records for Massachusetts, and *Myrmica scabrinodis*, a new record for the United States.

**Table 2:** Island-by-island occurrence data for ants in the BHI from the 2005–2009 ATBI.

	Bumpkin	Calf	Grape	Gr. Brewster	Langlee	Ragged	Snake	Spectacle	Thompson	Worlds End
**Amblyopone pallipes**	1	2	2	2	5	1	0	0	12	2
**Anergates atratulus**	0	0	0	0	0	0	0	0	1	0
**Aphaenogaster fulva**	0	1	0	0	0	0	0	0	0	0
**Aphaenogaster rudis Complex**	24	27	20	11	51	24	0	0	84	90
**Brachymyrmex depilis**	3	0	5	6	13	1	0	1	0	2
**Camponotus americanus**	0	0	0	0	0	0	0	0	0	1
**Camponotus caryae**	0	0	0	0	1	0	0	0	1	2
**Camponotus nearcticus**	1	2	1	0	2	12	0	0	3	3
**Camponotus novaeboracensis**	0	1	0	0	0	1	0	0	0	0
**Camponotus pennsylvanicus**	6	0	23	0	19	45	0	3	20	45
**Crematogaster cerasi**	0	1	13	16	8	22	4	4	11	17
**Crematogaster lineolata**	13	1	1	12	11	24	7	11	27	4
**Formica dolosa**	0	0	0	0	0	0	0	0	0	4
**Formica incerta**	0	1	14	0	5	2	10	8	20	10
**Formica lasioides**	0	0	1	0	0	0	0	0	0	0
**Formica neogagates**	1	0	0	0	3	0	0	0	0	6
**Formica subsericea**	25	0	12	0	9	32	0	0	19	27
**Lasius alienus**	9	1	34	5	4	1	2	27	18	7
**Lasius claviger**	0	0	1	0	0	0	0	0	1	1
**Lasius interjectus**	2	0	3	1	0	0	0	0	2	0
**Lasius latipes**	0	0	4	0	0	0	0	0	1	0
**Lasius nearcticus**	0	0	0	4	4	1	0	0	0	7
**Lasius neoniger**	8	1	13	20	3	0	9	12	61	6
**Lasius pallitarsis**	0	11	5	8	0	0	1	2	6	3
**Lasius subglaber**	0	0	0	0	1	0	0	0	0	0
**Lasius umbratus**	1	0	3	0	2	0	0	0	5	5
**Monomorium emarginatum**	0	0	0	0	0	0	6	2	2	0
**Myrmecina americana**	0	0	0	0	0	0	6	0	2	0
**Myrmica “sculptilis”**	3	1	14	0	2	0	0	0	0	25
**Myrmica “smithana”**	9	0	5	1	0	0	0	0	0	14
**Myrmica americana**	0	0	0	0	0	0	0	2	1	2
**Myrmica fracticornis**	1	0	16	0	0	0	1	6	2	1
**Myrmica pinetorum**	0	0	2	0	1	0	0	0	1	1
**Myrmica punctiventris**	9	0	30	8	6	34	0	0	20	30
**Myrmica rubra**	19	54	15	72	4	3	26	6	57	15
**Myrmica scabrinodis**	0	0	5	0	0	0	1	9	6	1
**Nylanderia flavipes**	0	0	0	0	0	0	0	0	59	0
**Ponera pennsylvanica**	6	12	17	10	9	23	13	2	49	6
**Prenolepis imparis**	4	2	29	3	22	5	15	1	64	18
**Protomagnathus americanus**	0	0	1	0	0	0	0	0	0	0
**Pyramica metazytes**	0	0	0	0	0	0	0	0	1	0
**Solenopsis molesta**	11	0	18	11	2	5	3	0	36	1
**Stenamma brevicorne**	1	14	17	28	2	3	13	2	26	12
**Stenamma impar**	2	6	20	0	6	9	3	0	12	5
**Stenamma schmitti**	1	1	12	0	2	4	0	0	1	2
**Tapinoma sessile**	14	6	32	30	6	9	12	16	12	17
**Temnothorax ambiguus**	1	13	18	19	0	3	11	14	8	5
**Temnothorax curvispinosus**	6	2	51	1	11	9	15	4	14	9
**Temnothorax longispinosus**	0	0	4	2	13	21	1	2	12	8
**Temnothorax schaumii**	0	0	0	0	0	0	0	0	0	2
**Tetramorium caespitum**	9	32	48	59	9	25	17	68	56	9

*Notes:* “Occurrence” defined as appearance in any single collecting event at each island. Total number of occurrences is 3,311.

**Table 3:** Observed number of collected species, and estimates of total active species, mean species collected per plot, and spatial and temporal turnover for ants in the BHI.

Island	observed species		estimated active species		species per sampling event		turnover	turnover
	(*S_obs_*)		(*S*)				(space)	(time)
	year	week	year	week	year	week	year	year
**Bumpkin**	27	10.38±8.11	30.33±2.08	29.32±8.08	2.53±3.34	3.60±2.11	7.54±2.92	0.73±0.10
**Calf**	22	6.07±3.12	27.38±3.82	12.64±2.96	1.24±1.41	1.45±0.54	9.85±3.20	0.61±0.11
**Grape**	36	10.76±7.35	37.70±1.33	23.87±5.98	1.89±2.37	2.18±0.83	9.58±2.79	0.49±0.17
**Gr. Brewster**	22	9.73±4.41	23.22±1.17	23.32±9.38	1.70±1.94	2.02±0.64	9.03±3.07	0.66±0.12
**Langlee**	30	11.62±6.12	32.98±1.32	18.75±5.92	2.06±2.72	2.41±1.27	9.84±8.79	0.53±0.13
**Ragged**	25	9.33±6.56	26.13±1.22	19.31±3.44	2.31±2.04	2.61±0.71	6.72±1.69	0.66±0.18
**Snake**	21	9.00±4.82	21.30±0.69	19.06±3.48	3.00±2.78	3.38±1.35	5.24±1.70	0.54±0.20
**Spectacle**	21	6.57±3.03	23.03±1.22	14.30±2.25	1.20±1.60	1.60±0.41	9.60±1.86	0.61±0.16
**Thompson**	38	12.14±6.93	39.36±1.54	21.62±4.45	1.38±2.04	1.84±0.82	13.80±10.56	0.58±0.10
**Words End**	39	10.33±7.49	42.49±2.03	25.51±4.73	1.66±2.00	1.96±1.00	10.73±4.12	0.40±0.17
**total BHI**	51	25.09±7.00	51.08±1.00	30.72±4.36	1.69±2.16	2.14±1.07	9.76±5.74	0.56±0.17

*Notes:* Mean estimates ±1SD. *S_obs_* is observed number of species collected during sampling, whereas *S* is output from rarefaction-based asymptotic estimate of total active ant species. Species per sampling event measured in mean number of species collected per sampling event. Turnover in space is measured as sampling event heterogeneity, or average fraction of total island diversity *S* found in a single sampling event. Turnover in time is measured as Bray-Curtis dissimilarity between sequential sampling weeks.

### No significant biases in inventory or species activity metrics

**Figure 2:**
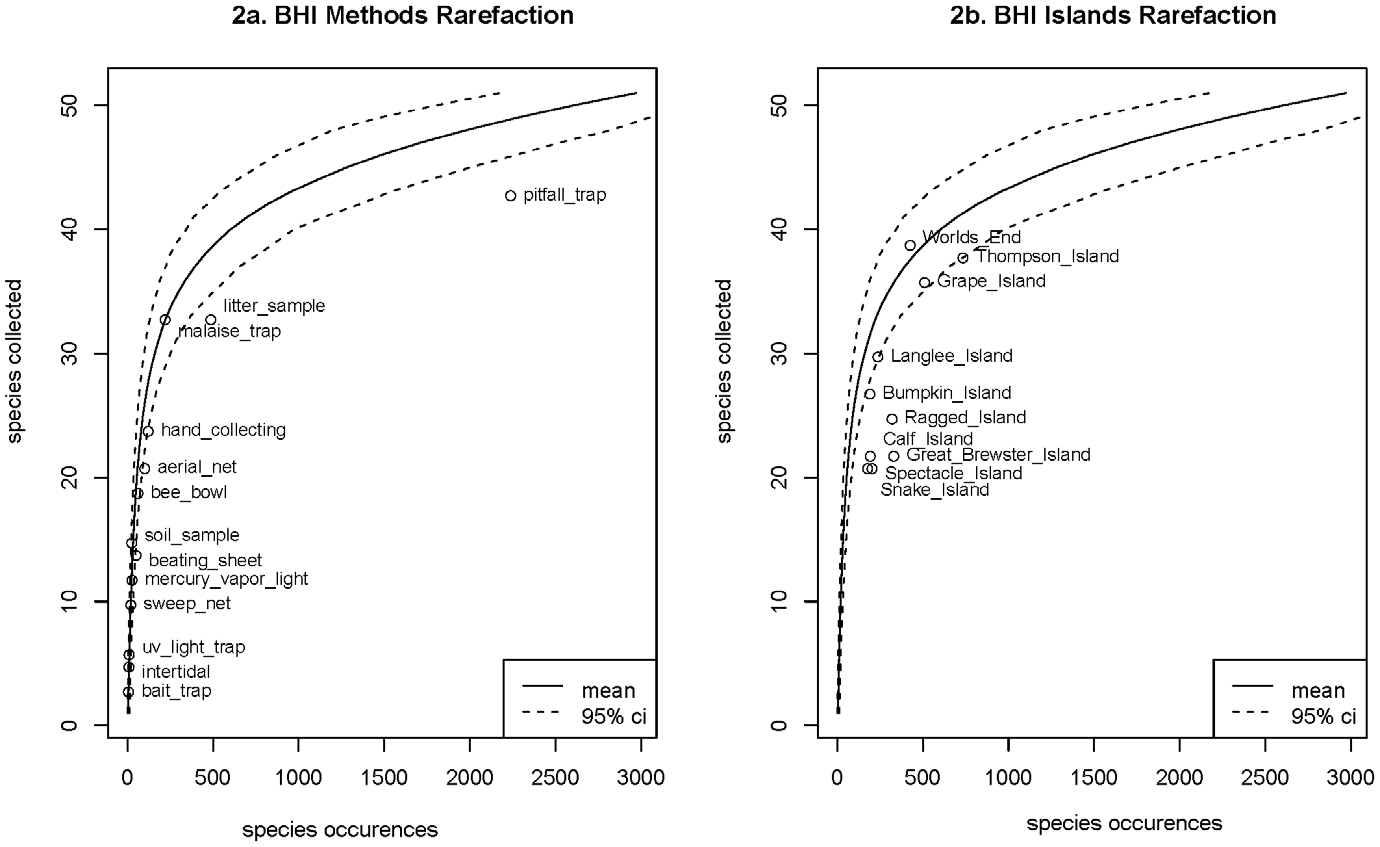
Rarefaction curves, in which total sampling occurrences of ant species from the ATBI were sampled without replacement, and resulting number of collected species was plotted against the number of species occurrences. Solid lines represent mean value of 1,000 simulations, dashed lines represent 95% confidence interval. Compares expected number of species collected between sampling methods (2a) and islands (2b) based on species abundance. Plotting species against number of individuals collected yields identical results.

The shallow slope of the rarefaction curve for total species collected on the ten islands in the BHI that we sampled suggests that we have collected most ant species that are present, and that additional sampling effort would be unlikely to collect many new species (Figure 2). This is supported by our asymptotic estimate, which predicts total number of ant species present on the BHI of 50.76 (±1.03 SD).

For most sampling methods, the number of species captured by each sampling methods fell within the 95% confidence interval of our rarefaction simulations (Figure 2a). While pitfall traps consistently under-sampled ant species, they accounted for roughly the same fraction of sampling on each island (0.334±0.037SD) and likely did not contribute to a particular bias on any island. Repeating our estimation procedures using only data from pitfall collections yielded much coarser, but qualitatively similar, results (See Supporting Information S2). Sampling for most islands, on the other hand, fell outside of the 95% confidence interval for expected number of species, suggesting significant differences in species composition between islands (Figure 2b). Nonetheless, rarefaction curves and the corresponding asymptotic estimates of the number of active species on each island suggest that we successfully collected most species present on each island, and that there was little sampling bias between islands or methods (Table 3).

### Observed number of species does not change for season, disturbance, or peninsulas

Based on our analyses, we can discount three potentially confounding factors. First, our analyses of seasonal patterns suggest that temporal differences in species composition is a stochastic rather than climatological process, and that differences in the number of species we collected in spring, summer, and fall are a result of changing sampling intensity, rather than of ant ecology. Ant species in New England appear to have more or less the same “active” season (See Supporting Information S3). Second, we also found no significant differences in the number of active ant species resulting from disturbance, neither at the level of islands nor at the level of individual plots, with the single exception of Spectacle Island, which was recently capped under more than a meter of clay and earth when it was converted from a landfill in 2006 (See Supporting Information S4). Finally, repeating our analyses without Worlds End to account for its connection to the mainland, we found no differences in the significance of our ANCOVAs, except in the association of area and temporal turnover, which was slightly diminished (See Supporting Information S7).

### Observed number of species depends on timescale considered

**Figure 3:**
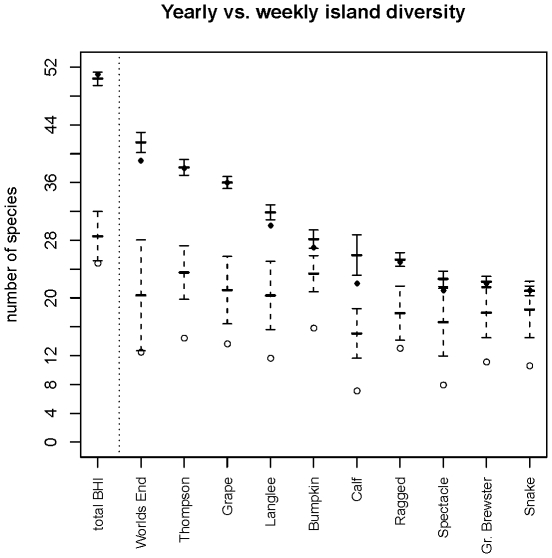
Estimated mean number of active species (±1SD) by island for total sampling season from asymptotic function fitted to rarefaction curves. Circles show observed number of species collected. Solid/filled show total sampling events, whereas dashed/open show the average week of sampling. Islands are ordered from largest to smallest number of estimated species.

Based on our asymptotic estimates, we found a striking contrast between the total number of species that could be collected on islands over the course of a sampling season and the actual number that could be collected at any one moment in time (Figure 3). All islands showed significantly lower estimated active species over the course of the average week than over the entire year – some by almost 50%. While the asymptotic estimate was in all cases higher than the observed number of species, smaller sample size for weekly estimates led to higher variance, and a stronger under-sampling among observed species. Our asymptotic estimates were not significantly different from those generated by EstimateS, though our asymptotic method always had much smaller standard error, likely as a result of the large number of single and double occurrences of species in our data, which Chao's method uses to estimate species richness (See Supporting Information S2).

### Diversity metrics are consistent across time, different among islands

Comparing the number and identity of species between islands and through time, we sought to explain both the magnitude and cause of differences in ant diversity between islands. We found significant differences among islands in the estimated number of active species, average number of species captured per sampling event, sampling event heterogeneity, and temporal turnover of islands. These differences are associated with island size and isolation from the mainland. As predicted by the classical theory of island biogeography, the number of active species on an island was positively correlated with island area (p = 0.03, adjusted r^2^ = 0.38), and negatively correlated with island isolation (p = 0.02, adjusted r^2^ = 0.43). However, because standard log/log transformations did not reveal significant correlations (p≫0.05), and due to the weak signal in both linear models, we divided both area and isolation into two levels of “small and large”, “near and far”, for all following analyses.

**Figure 4:**
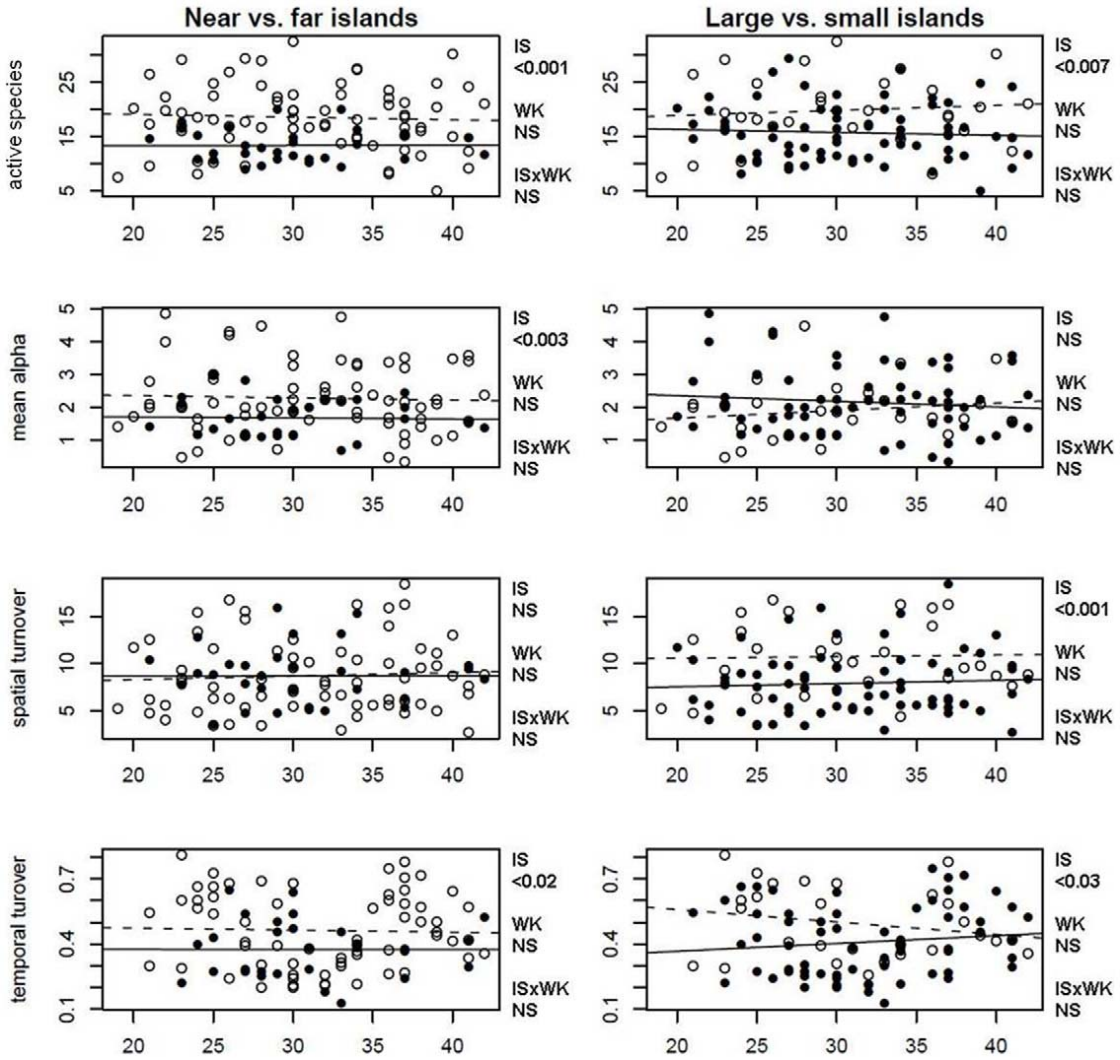
ANCOVA of island area and isolation per week against estimated number of active species, mean species per sampling event (alpha), spatial turnover, and weekly temporal turnover. Open circles show near and large islands, closed show small and far. “Near” islands are 0–1.65 km and “far” islands are 1.65–3.29 km from the nearest mainland; “small” islands are 0–0.54 km^2^ in terrestrial area above the high tide mark and “large” islands are 0.54–1.08 km^2^. “IS” shows the test statistic for differences between islands, “WK” between weeks, and “ISxWK” the interaction effect.

We found no significant differences in our four diversity parameters for samples taken across time on the same island (Table 3; Figure 4; see S6, S7 in the Supporting Information for ANOVA tables). As such, the number of active species and rates of turnover appear to remain constant through time on each island. Additionally, there were no significant interaction effects between island isolation or island size and week of sampling. However, diversity metrics did differ based on island's biogeographies. Estimated total number of active species was significantly higher on islands near the mainland than far from it (p<0.001), and higher on large than small islands (p<0.007). Mean species collected per sampling event was significantly higher on near islands than on far (p<0.003), but not large islands. Spatial turnover was not significantly different between near and far islands, but was significantly higher on large islands than on small islands (p<0.001). Finally, temporal turnover was significantly higher for near islands than for distant ones (p<0.02), and significantly higher for large islands than for small islands (p<0.03). Our analysis thus revealed that differences in island area and isolation are indeed associated with differences in ant diversity. Moreover, there is no significant difference from week to week in any of our diversity metrics. Though the identity of species collected changed greatly over time, the number of active species in our sampling areas remained constant.

### Ant species cycle through time, and are affected by disturbance

Our analysis revealed very few multi-species ant communities on the BHI (See Supporting Information S3.2). For the community matrix based on the entire ATBI dataset, the stable state distribution suggests that over 95% of observed community states through time can be described as the result of “communities” of a single species. That is, though there are generally more than one species present per sampling event, the vast majority of non-random transformations through time are between individual species, not between assemblages of multiple species.

**Figure 5:**
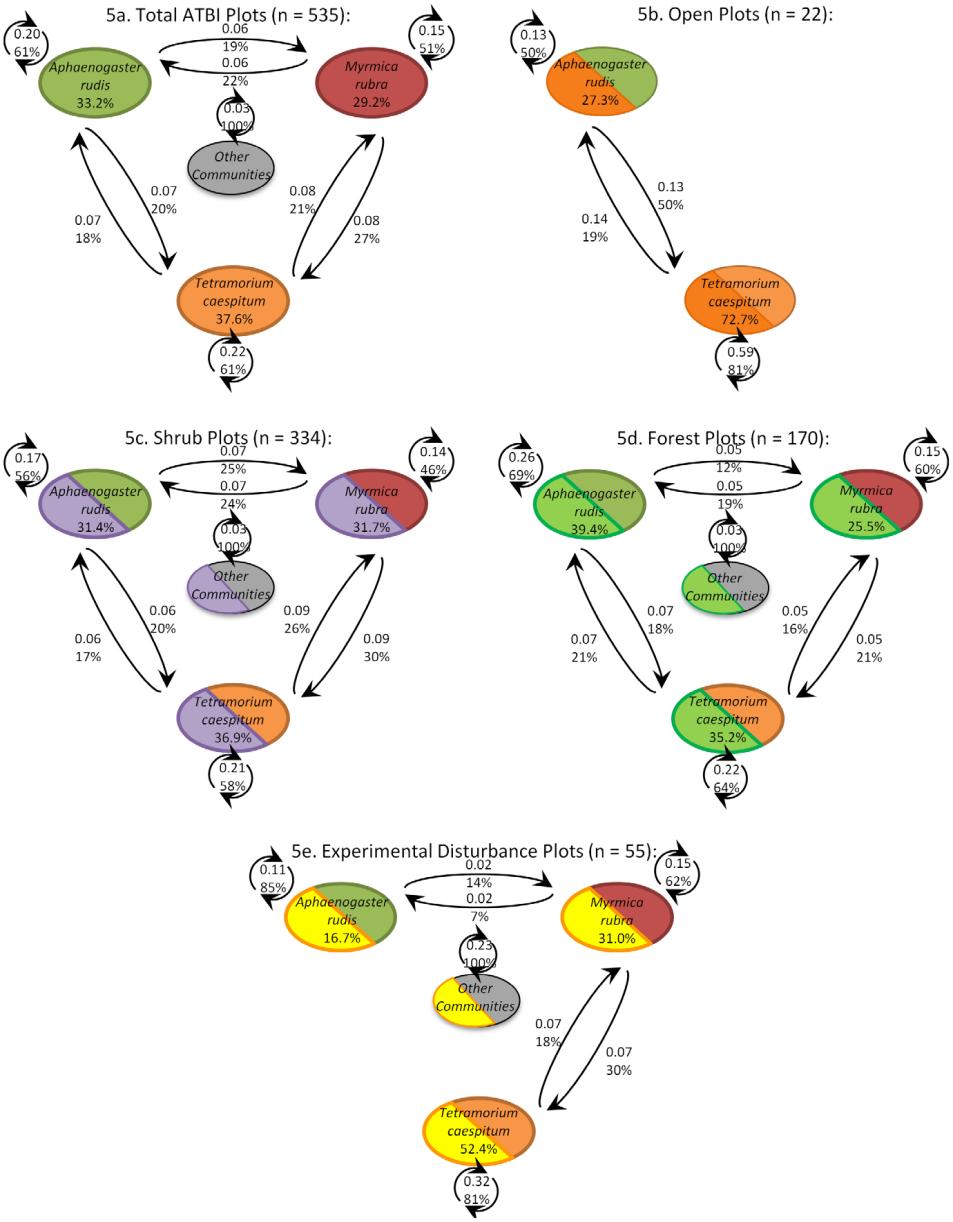
Simplified transition probability matrix for four species classes based on data from the 2005–2009 ATBI of ants on the BHI. Sample number, n, shows number of transitions between states used to compute each matrix. Each time step is approximately two weeks. Fractions above transitions show “mass flux” of system, or fraction of total transitions moving between the indicated states. Percent above transitions shows fraction of each state following a particular transition (e.g. A→B shows probability that A progresses to B over one unit of time). Percent under species name shows predicted stable state distribution from Eigen decomposition.

In all of our community tables, the native ant species *Aphaenogaster rudis*, and the exotics *Myrmica rubra*, and *Tetramorium caespitum*, accounted for 40–60% of observed community states over time. Based on this, we categorized these three species as the system's dominant species, and constructed a reduced transition matrix focusing on them, and collapsing all other species into a common fourth column. The modified transition matrices (Figure 5a–e), accounting for these three species and “all other communities” as the only four states in the system, revealed significant differences in the species composition, and species dynamics, of ants on the BHI depending on disturbance and habitat.

In comparison to the dynamics of the total ATBI plots (Figure 5a), open plots (Figure 5b) displayed highly modified transition (p<0.01) and state (p<0.01) structure, with a total absence of *M. rubra*, and *T. caespitum* taking up over 70% of the stable state distribution. Likewise, plots from the disturbance experiment (Figure 5e) contained fewer instances of the native forest ant *A. rudis* and more of the exotic species *M. rubra* and *T. caespitum* (p = 0.02), and transitions between states were significantly reduced (p<0.01). However, all three species were more likely to remain present in the plot through time, rather than be replaced by a different group, thus exhibiting less turnover and more community stability within the invaded state. Shrubby habitats (Figure 5c) and forested habitats (Figure 5d), on the other hand, were not significantly distinct from the pooled dataset in their transition probabilities (p = 0.33, p = 0.17) nor in their stable state distribution (p = 0.71, p = 0.33).

## Discussion

Our findings suggest that the number of active ant species on islands in the BHI remains relatively constant through time, and that its magnitude is significantly determined by the island's biogeographic factors. Much of the difference in the number of active species among islands can be explained by an island's isolation from the mainland and its size. However, our results also support the hypothesis that the actual magnitude of this number depends on the timescale under consideration. That is, many different species of ants appear to share the same *space* on islands at different *times*.

All three diversity metrics that we used varied based on island biogeography. Average sampling event diversity depended largely on island proximity to the mainland, and was significantly higher on near islands than far islands. Spatial turnover, on the other hand, depended on size, and was significantly higher on large islands than small. These patterns are readily explainable following classical island biogeography, likely resulting from higher overall species richness on near islands, and various factors associated with increased area, such as increased and more heterogeneous niche space and decreased rates of local extinction [2], [6], [52].

The observed patterns of temporal turnover pose a particularly interesting quandary. Week for week, we collected the same number of species in plots, but the identity of those species continuously changed. Partially, this could be due to local immigration and extinction of species among islands, but this is inconsistent with ant natural history since colony dispersal is such a slow process [53]. Instead, we must assume that most of the species sampled over the course of the year are present on the island at *some* level for the entire season. Particularly, we can imagine that if a species does not have workers actively foraging in the regions that we are sampling, the species will appear to “disappear” from the island for a time [43].

Differences in temporal turnover across island biogeography hint towards a mechanism behind these changes in ant activity. Large islands and islands close to the mainland both display significantly higher temporal turnover between weeks than small or isolated islands. While this could be the result of higher island species richness and sampling omission, such an explanation would require remarkably homogeneous community structure among all groups of species because the diversity of individual sampling events varies so little between weeks. A more likely explanation is that that higher species richness on near and large islands leads to higher levels of competition between species, and therefore variations in length of time that particular species are active throughout the year and the length of time that species occupy any single plot. Certainly, competition plays an important role in the formation of ant communities [31], [32], [34], [35], [54].

Our community dynamics model shows how interactions between species might lead to these observed changes. Our model focuses only on the three most common ant species: the native species *A. rudis*, and the exotic species *M. rubra* and *T. caespitum*. However, it illustrates the general patterns that most species could follow. At the average sampling site, all three common species were collected with more or less equal frequency. However, the observed “cycles” at any single sampling site were highly predictable. Depending on the species that were present one week, the probability of collecting each species next sampling period changed considerably. Generally, species reinforced self-occupancy, increasing the probability of their own persistence at the cost of the other two common species. This pattern varied surprisingly little among habitat types.

Experimentally disturbed plots and open plots, which were regularly mowed, were subject to significantly altered residency and transition patterns. Both cases led towards simpler communities with fewer transitions and a significant tendency towards exotic species. In disturbed plots, there was a sharp decrease in the abundance of *A. rudis* with a corresponding increase in the abundance of *M. rubra*. Similarly in open plots, *A. rudis* decreased in abundance in favor of *T. caespitum*, whereas *M. rubra* was not collected at all. In both cases, the model predicts that this is the result of a change in transition probability. Though there are overall fewer transitions between species states, when they do occur, they tend to favor the two exotic species.

Biologically, this tendency in more disturbed plots makes good sense [27]. *A. rudis*, generally speaking, prefers moist and vegetated environments, and often builds large, active nests in these regions, which would certainly compete with the nests of other species. However, it does less well in open environments. *T. caespitum* and *M. rubra*, on the other hand, are tramp species that do best in dryer and sandier regions. *M. rubra* in particular can be quite aggressive, and could easily out-compete other species in favorable environments [55].

Similar patterns should hold among less dominant species. Moreover, the disappearance of a species from a plot need not signal that it has been locally extirpated. Many species, such as those in the genus *Temnothorax* or *Solenopsis* are well-known for their ephemeral nesting habits. Because their small nests in structures such as hollow twigs or acorns are often disturbed, they move frequently on the scale of several meters [56], [57]. Even larger nests, such as those in the genus *Aphaenogaster*, have been shown to move on the scale of weeks, in response to environmental changes or heavy parasite loads [58]. Additionally, for any nest the number of workers foraging can vary greatly through time, and in many species nests can remain entirely closed in times of distress, without sending out foragers at all. Combined, the relocation of ants' nests and reclusion of nests throughout the year could lead to varying species composition across sampling sites through time.

Our findings are novel and exciting for several reasons. Across large scales, the results from this study accord well with the classical theory of island biogeography. On each island, we find that overall species richness, sampling event diversity, and patterns of spatial and temporal turnover depend on islands size and isolation from the mainland. On smaller scales, we find a constant number, but continuously changing cast, of species at plots throughout the sampling season. The diversity observed at any single moment in space and time, therefore, is likely due to a combination of large-scale biogeographic processes and the small-scale effects of interspecific competition and nest relocation. These two processes mirror one another quite nicely. Just as species shuffle among islands on the scale of years following the laws of island biogeography, species shuffle among plots within individual islands following the laws of interspecific competition.

Resulting from these two scales of species sorting, we have also demonstrated that almost half of the Massachusetts ant fauna, including four species new to the state (S. Cover, personal communication) and one new to the United States (A. Francoeur, personal communication), can be collected in a relatively small, isolated, and heavily utilized urban park. Based on the peculiarities of the BHI, larger mainland parks should, if anything, have even higher ant diversity. This finding is not trivial, and has strong implications for the conservation of species in a world that is increasingly characterized by fragmented islands of habitat surrounded by largely human-dominated landscapes [59], [60]. Such “patches” of conservation may well be able to harbor significant populations of ants and other arthropods, even in heavily urban and disturbed environments.

## Supporting Information

Supporting Information S1 Ants of the Boston Harbor Islands, Boston MA.
(DOCX)

Supporting Information S2 Comparison of Diversity Metrics.
(DOCX)

Supporting Information S3 Nonrandom Temporal Species Associations.
(DOCX)

Supporting Information S4 Effects of Disturbance.
(DOCX)

Supporting Information S5 BHI Habitat Maps.
(DOCX)

Supporting Information S6 ANOVA tables (Worlds End included in analysis).
(DOCX)

Supporting Information S7 ANOVA tables (Worlds End NOT included in analysis).
(DOCX)

Supporting Information S8 Boston Harbor Island ant sample data (available online at http://insects.oeb.harvard.edu/boston_islands/).
(DOCX)
