# Supplementary material for: The Effects of Biogeography on Ant Diversity and Activity on the Boston Harbor Islands, Massachusetts, U.S.A
Source: PLoS One. 2011 Nov 29;6(11):e28045. doi: 10.1371/journal.pone.0028045 (PMC3226633; doi:10.1371/journal.pone.0028045)

**S1. ANTS OF THE BOSTON HARBOR ISLANDS, BOSTON MA**

**Dolychordinae**

*Tapinoma sessile* (Say), 1836

**Formicinae**

*Brachymyrmex depilis* Emery, 1893

*Camponotus americanus* Mayr, 1862

*C. caryae** (Fitch), 1855

*C. nearcticus* Emery, 1893

*C. novaeboracensis* (Fitch), 1855

*C. pennsylvanicus* (De Geer), 1773

*Formica dolosa* Buren, 1944

*F. incerta* Buren, 1944

*F. lasioides* Emery, 1893

*F. neogagates* Viereck, 1903

*F. subsericea* Say, 1836

*Lasius alienus* (Förster), 1850

*L. claviger* (Roger), 1862

*L. interjectus* (Mayr), 1866

*L. latipes* (Walsh), 1863

*L. nearcticus* Wheeler, 1906

*L. neoniger* Emery, 1893

*L. pallitarsis* (Provancher), 1881

*L. subglaber* Emery

*L. umbrat*us (Nylander), 1846

*Nylanderia flavipes^E^* (F. Smith), 1874

*Prenolepis imparis (*Say), 1836

**Myrmicinae**

*Anergates atratulus**^,^*^E^* (Schenk), 1852

*Aphaenogaster fulva* Roger, 1863

*A. rudis* complex

*Crematogaster cerasi* Fitch, 1855

**Myrmicinae (ct’d)**

*C. lineolata* (Say), 1836

*Monomorium emarginatum* DuBois, 1986

*Myrmecina americana* Emery, 1895

*Myrmica* americana Weber, 1939

*M. fracticornis* Forel, 1901

*M. pinetorum* Wheeler, 1905

*M. punctiventris* Roger, 1863

*M. rubra^E^* Linnaeus, 1758

*M. scabrinodis****^,E^* Nylander, 1846

*M.* undescribed species [*“sculptilis”*]

*M.* undescribed species [*“smithana”*]

*Protomagnathus americanus* (Emery), 1895

*Pyramica metazytes** Bolton

*Solenopsis molesta* (Say), 1836

*Stenamma brevicorne* (Mayr), 1886

*S. impar* Forel, 1901

*S. schmitti* Wheeler, 1903

*Temnothorax ambiguus* (Emery), 1895

*T. curvispinosus* (Mayr), 1866

*T. longispinosus* (Roger), 1863

*T. schaumii* Roger, 1863

*T. caespitum^E^* (Linnaeus), 1758

**Ponerinae**

*Amblyopone pallipes* (Haldeman), 1844

*Ponera pennsylvanica* Buckley, 1866

* New record for Massachusetts

** New record for United States

*^E^* Exotic to North America

**Natural history data on new records**

We collected four ants which are new records for New England: *Anergates atratulus*, *Camponotus caryae*, *Myrmica scabrinodis,* and *Pyramica metazytes*. Of these, *Myrmica scabrinodis* is likely also a new record for North America.

*A. atratulus* and *P. metazytes* were each collected in only one sampling event, and we therefore cannot provide much useful natural history information on their natural histories. *A. atratulus* is an obligate nest parasite of *Tetramorium caespitum*. Though it rarely leaves its host nest and is seldom collected, it is likely relatively common in its native European and introduced North American ranges. Our specimen was a single virgin queen collected in a pitfall trap in a salt marsh on Thompson Island in late August. *P. metazytes* has appeared in collections across the southern and western United States. Our specimen was a lone worker collected in a leaf-litter sample taken from a deciduous forest, also on Thomson Island, in mid-September.

*C. caryae* has been collected primarily in sites farther south than Massachusetts, though recent collecting in New York State has led to the capture of several specimens (G. Alpert, personal communication). We collected four workers between June and October on three islands: Langlee, Thompson, and Worlds End. It is likely that this species is present in low numbers throughout Massachusetts and New England, but as it is a generally arboreal species that often nests in hickory trees (G. Alpert, personal communication), it is rarely collected. Collection sites in the BHI included oak/hickory deciduous forests on Langlee (pitfall trap) and Thompson (hand collecting), and a an old field on Worlds End (malaise trap).

*M. scabrinodis* is relatively common on the BHI, occurring is some 23 sampling events on five islands. Most collections occurred in old fields between June and August using a combination of pitfall traps and leaf-litter samples. However, a few specimens were found later in the autumn. Again, most collections were made using pitfall and leaf-littler traps, though hand collecting and malaise traps each yielded one sample.

*M. scabrinodis* is endemic to Europe, and to the best of our knowledge has never been collected in the United States prior to our study. Many historical records of ants in Massachusetts and New England list *M. scabrinodis*. However, based on records at the Harvard MCZ and Cornell Insect Collection, these identifications are likely the erroneous result of attempts to identify North American ants using European keys. *M. scabrinodis* is taxonomically quite similar to two undescribed *Myrmica* species, *M.* “*sculptilis*” and *M.* “*smithana*”. If this is generally the case, the BHI likely represents a novel introduction of the species.


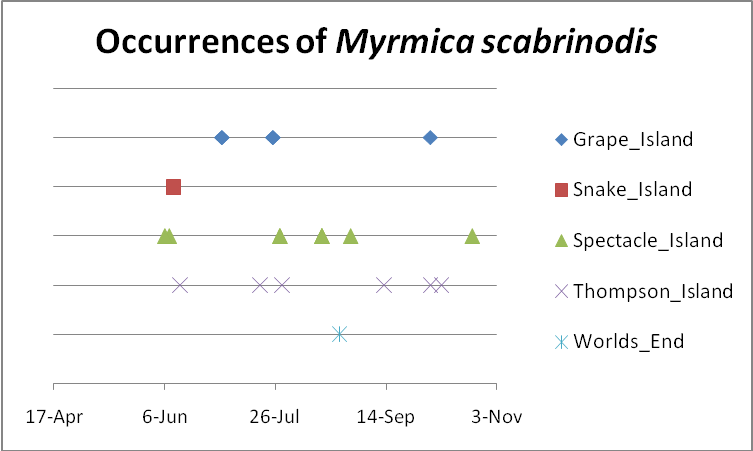

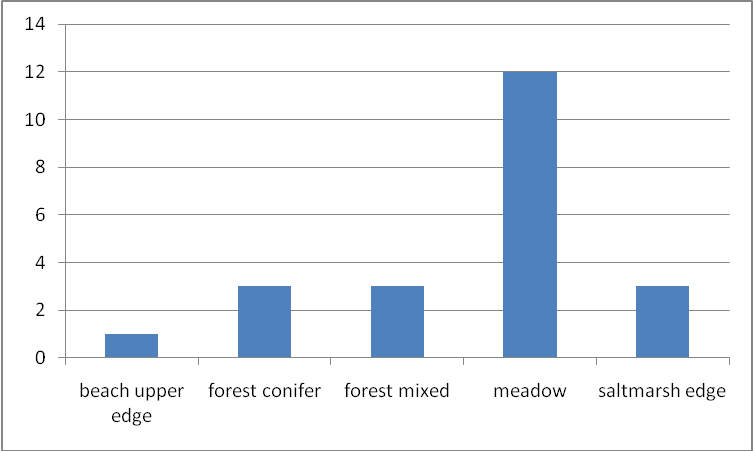

Supplement: Supporting Information S1 — Ants of the Boston Harbor Islands, Boston MA. (DOCX) [file pone.0028045.s001.docx]
