# Supplementary material for: The Effects of Biogeography on Ant Diversity and Activity on the Boston Harbor Islands, Massachusetts, U.S.A
Source: PLoS One. 2011 Nov 29;6(11):e28045. doi: 10.1371/journal.pone.0028045 (PMC3226633; doi:10.1371/journal.pone.0028045)
Supplement: Supporting Information S2 — Comparison of Diversity Metrics. (DOCX) [file pone.0028045.s002.docx]

**S2. COMPARISON OF DIVERSITY METRICS**

1. Rarefaction curve comparison


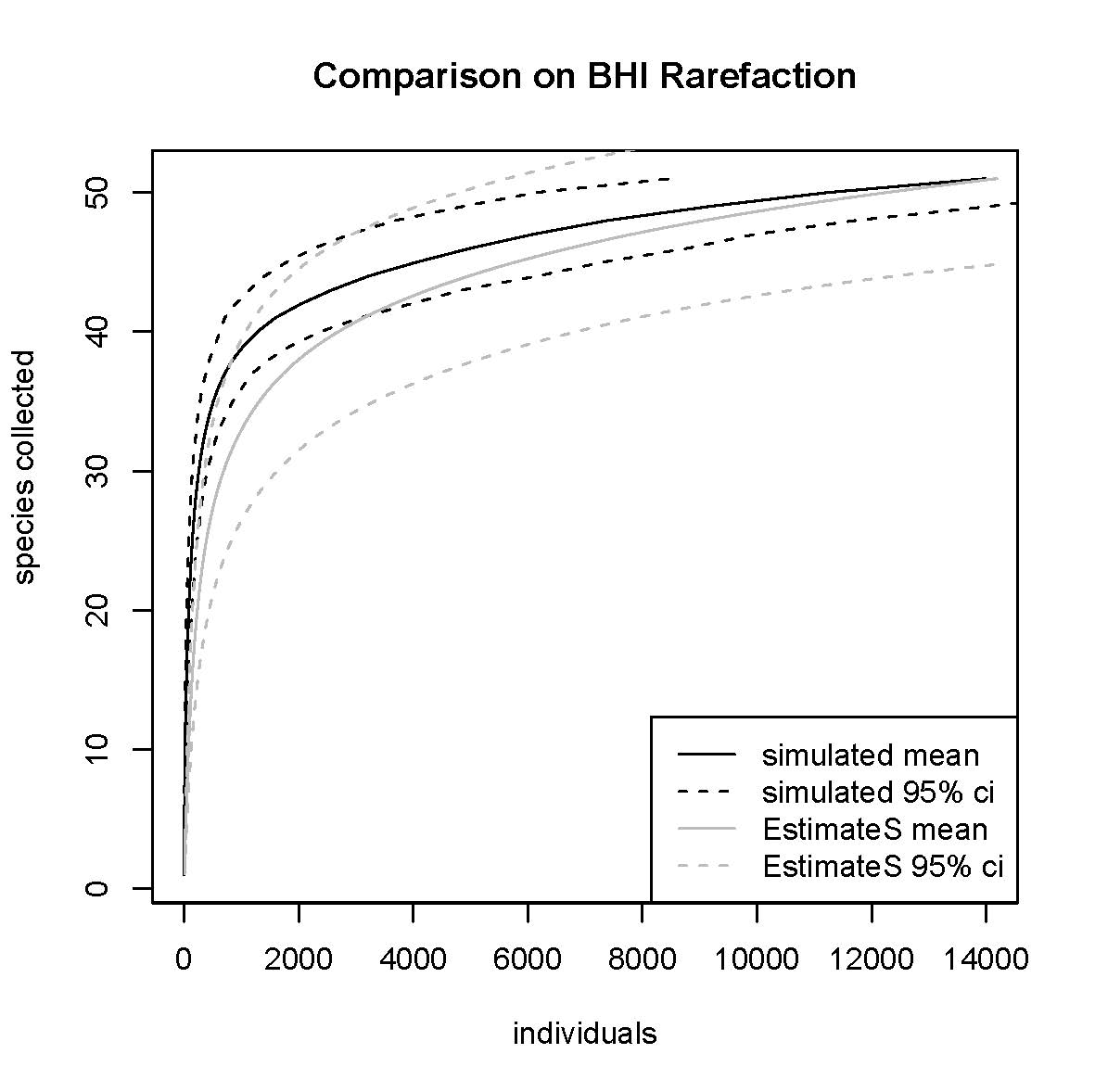


Fig. S2.1. In order to assess the accuracy of our rarefaction curves, we compared them to the sample-based MaoTau approximation in EstimateS version 8.2.0. We used identical datasets in each instance, and ran both simulations 1,000 times. The two curves are not significantly different and have similar mean values, though the MaoTau generated a wider confidence interval. We chose to use our rarefaction procedure for all estimates of active species, both because it appeared to generate a more precise estimate, and because it was easier to automate for our other tests.

2. Collecting method comparison


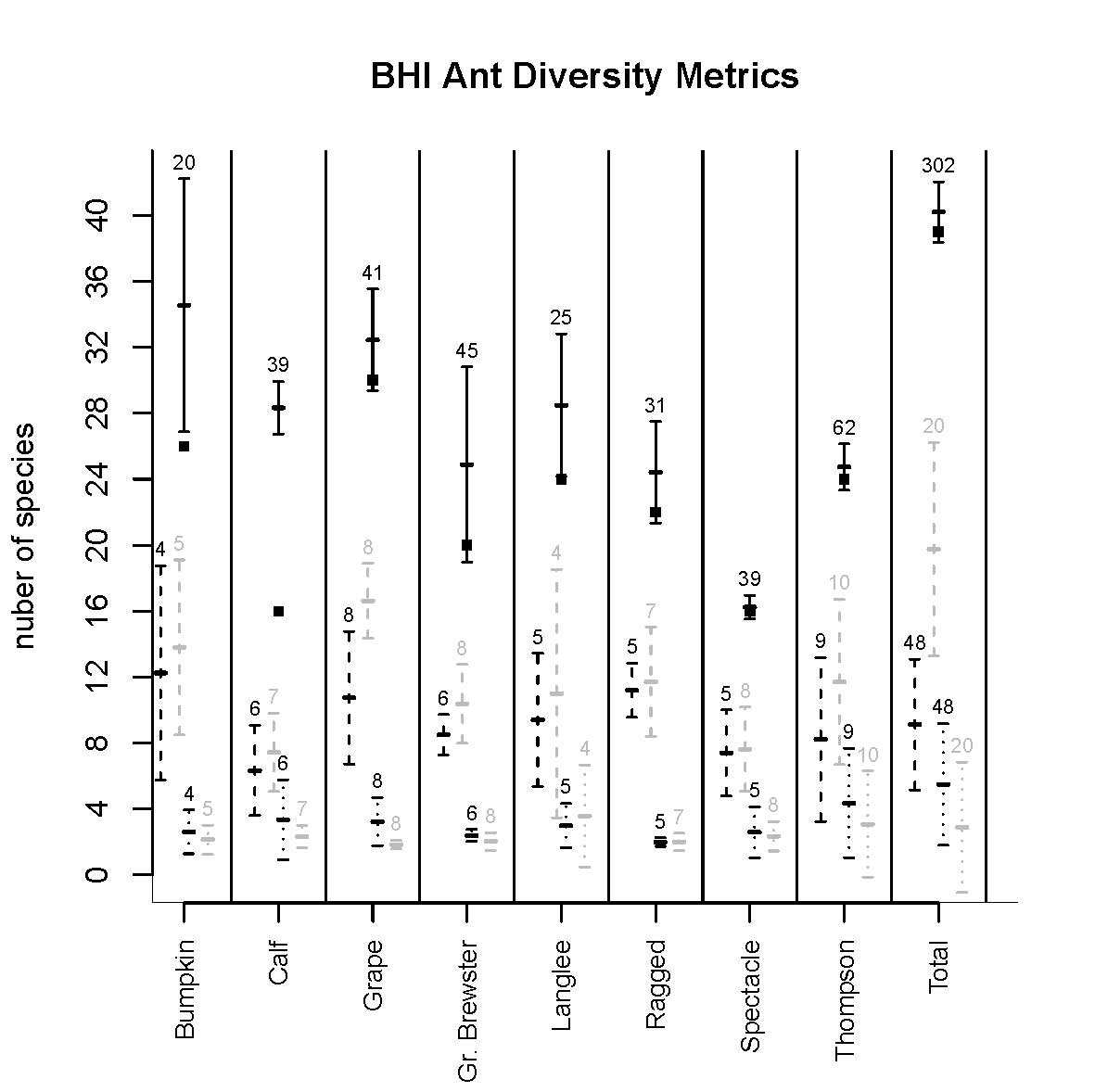


Fig. S2.2. The rarefaction curves presented in the main article show that the different sampling techniques implemented in the ATBI are not significantly biased in the number of species which they capture. A notable exception to this are pitfall traps. We repeated our estimates using only pitfall trap data. Solid lines show total estimated number of active species, dotted lines show mean sampling event diversity, and dashed lines show turnover. Squares show observed number of species. Black lines represent the dataset segregated by *space* (i.e. each event is a single spatial location), whereas gray lines show the dataset segregated by time (i.e. each event is a single week of sampling). All intervals show estimate ±1 SD. We found no significant differences in estimated number of active species, except for Spectacle Island, Thompson Island, and the pooled dataset. Because of the paucity of pitfall data, which resulted in very large confidence intervals for weekly diversity estimates, we chose to use the entire dataset rather than only data from pitfall traps.

3. Estimated number of species


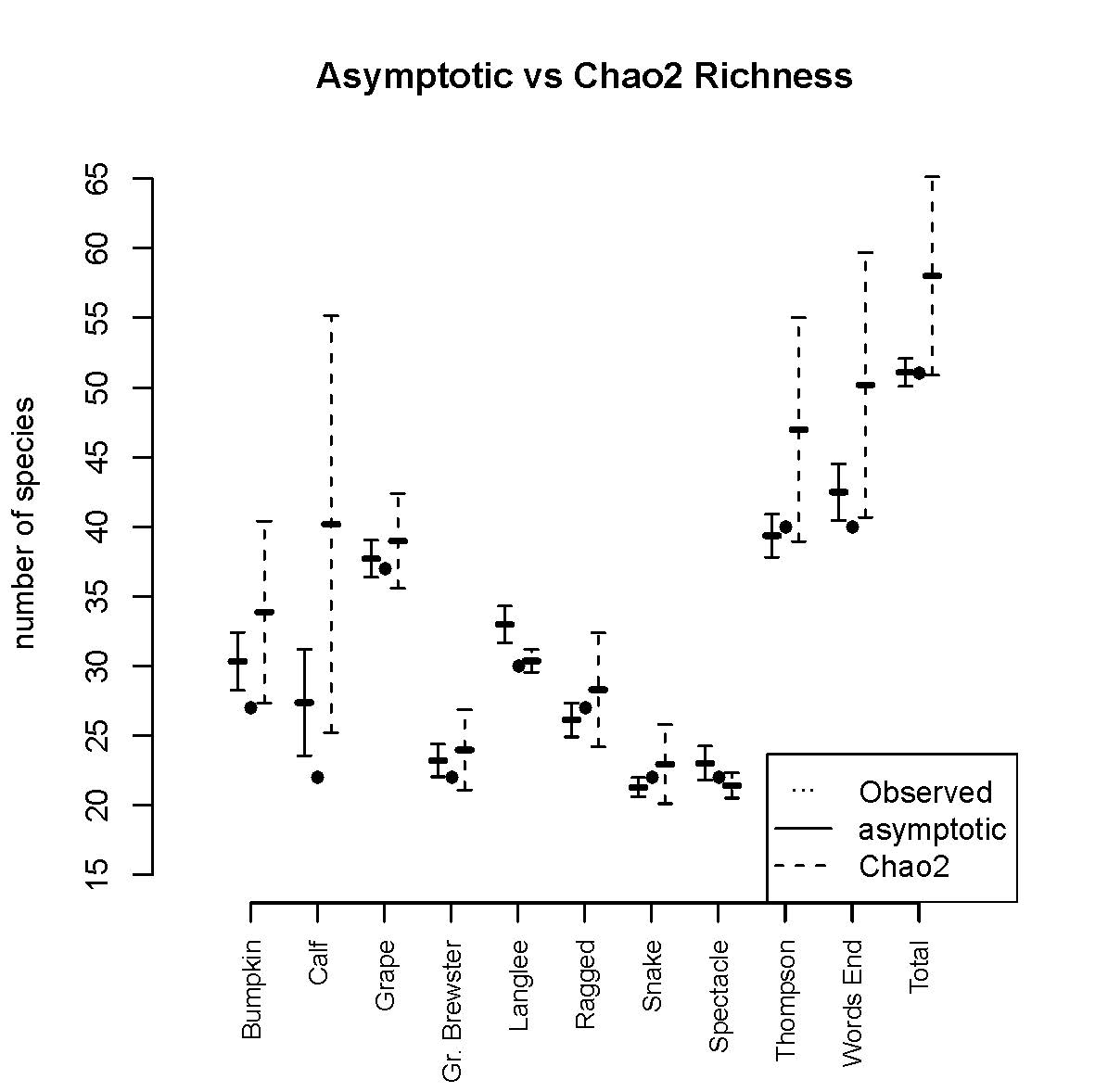


Fig. S2.3. To assess the accuracy of our asymptotic estimates, we repeated our analysis using EstimateS version 8.2.0 to compute the Chao II species richness estimate. This is a nonparametic estimate based on the number of collecting events, and single or double occurences of species. To generate confidence intervals (±1 SD), we allowed EstimateS to run 1,000 iterations on the same dataset used to generate our asymptotic estimates. With the exception of Langlee Island, Chao II estimates were not significantly different from our asyptotic estimates, though because of the large number of single or double occurences of species resulted in very large standard error for the prediction. Moreover, the Chao II estimate would occasionally require recalculation using adjusted formulas, making comparison between multiple estimates difficult.
