# Supplementary material for: The Effects of Biogeography on Ant Diversity and Activity on the Boston Harbor Islands, Massachusetts, U.S.A
Source: PLoS One. 2011 Nov 29;6(11):e28045. doi: 10.1371/journal.pone.0028045 (PMC3226633; doi:10.1371/journal.pone.0028045)
Supplement: Supporting Information S3 — Nonrandom Temporal Species Associations. (DOCX) [file pone.0028045.s003.docx]

**S3. NONRANDOM TEMPORAL SPECIES ASSOCIATIONS**

We utilized two methods to determine the origin of annual patterns in ant diversity in our dataset. First, we used empirical orthogonal analysis to test for temporal trends in the number of active species, and assess whether differences through the year represented an ecologically significant phenomenon, or was the result of sampling error. Second, we compared the association of individual species to time of year to assess whether species occurrences were randomly distributed thorough time. In neither case did we find a significant seasonal signal, i.e. all species show more or less the same phenological patterns.

1. Empirical orthogonal analysis

To determine overall temporal patterns of diversity, we used empirical orthogonal function analysis (EOA) – a method of principle component analysis (PCA) commonly used in climatology [1]. EOA takes a matrix of vectors representing temporal changes of some quantity over time – in our case, number of active species – and redefines the basis of the matrix to find linear combinations of time periods that maximize the variance in the quantity being measured [2]. Additionally, EOA calculates the relative magnitude and direction of this variation between species and islands respectively. EOA is a particularly useful method in this case because of robust methods to account for missing data. This allows for the comparison of the magnitude and significance of particular temporal patterns.

For our data, we compared changes in the weekly predicted number of active ant species using a 4-week running average. This revealed the temporal scales that corresponded to the largest variation in relative abundance of ant species. To test the significance of these trends, we constructed a 95% confidence interval using a null-model based on 1,000 Monte Carlo simulations. We used a modified version of the fixed-fixed algorithm [3] to construct occurrence matrices where the number of species sampled per week and per sample site remained the same, but the precise distribution of species through time was randomly reshuffled. We accomplished this based on an algorithm of our own design, generating random matrices using a Poisson number generator, and modifying randomly chosen row and column elements until the matrix row and column sums equaled those in our observed dataset.

EOA revealed a strong annual trend in number of ant species, explaining 62% of weekly variation in number of active ant species. The annual trend showed a period of about 25 weeks, starting in late May and ending in late November – roughly corresponding to the temperate summer months, and to the sampling season of the ATBI. However, subsequent comparison to our null model showed that this trend was not significant. Neither the period nor the amplitude of the trend was greater than expected by chance based on the timing and frequency of our sampling events. The annual cycle predicted by EOA can therefore be attributed to increased sampling effort during the middle of the year, rather than to an actual change in the number of species present on the BHI during our sampling season. Our analysis thus confirmed that there is no significant difference in the number of species active between May and November for ants in the BHI.

Our EOA analysis suggests that there is no significant annual trend in ant diversity or community structure during the summer months. As a result, our analysis implies that samples from early in the summer are comparable with those in mid-summer and late summer. This greatly simplifies data analysis, and is consistent with other studies’ findings for ant communities in New England [4,5], and consistent with previous studies, we find total number of ant species on a given island does not change much over the course of a sampling season [4,6-8].


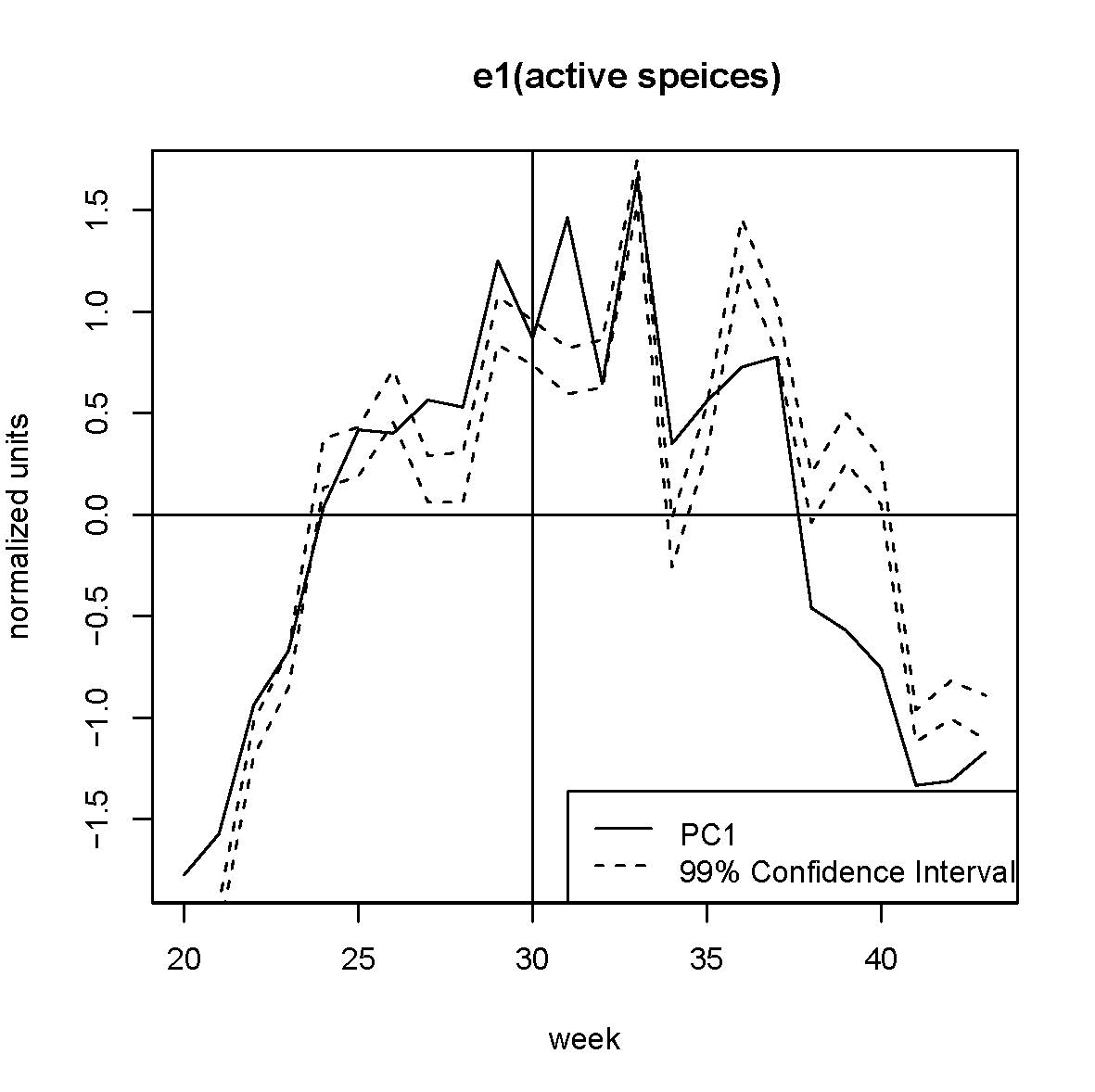


Fig. S3.1. First PCA vector, explaining 62% of variation in number of active species over time, from EOA procedure comparing predicted number of active ant species on the BHI to week of the year. Dotted line shows 95% confidence interval based on 1,000 Monte Carlo simulations of null dataset (see text).

2. Empirical Bayes method

Next, we assessed the extent to which weekly variations in island diversity were due to specific species’ phenologies. To do this, we used a modified method of the empirical Bayes approach [3]. This test adjusts for the type I error which is inherent in randomized analysis of large datasets. Rather than comparing C-scores, or relative spatial overlap between pairs of co-occurring species, we compared the fraction of species occurrences falling into any particular week. We then divided these fractions into 100 bins. We then generated 1,000 random communities using the “fixed-fixed” algorithm [3], which preserves both the number of occurrences per species and the number of species per sampling week.

Based on these simulations, we identified bins containing more species pairs than expected by chance, using both the mean-based and more conservative confidence-interval based Bayes approaches. Finally, using the pairs identified by the Bayes approach and the confidence intervals generated from our randomly simulated species communities, we identified specific instances in which species occurrences were significantly associated with particular weeks of the year. We then repeated the analysis with all singleton and doubleton species records (species that occurred in only one or two sampling events) removed from the dataset to account for potentially interfering factors in the analysis.

Analysis of specific species’ phenologies using empirical Bayes analysis based on the mean selection criterion revealed significant associations between 11 species and 12 weeks for analysis of the entire dataset, and 11 species and 11 weeks for analysis of the dataset with singletons and doubletons removed. The more conservative confidence interval-based test revealed only 3 species and 4 weeks for the total dataset and 3 species and 3 weeks for the curtailed one. Half of the species/week pairs, and five of the species, were common to both mean-based analyses. Likewise, half of the species/week pairs and half of the species were common to both confidence interval-based analyses. All species identified by this technique were relatively rare, making up only a handful of sampling occurrences. The analysis, and particularly the conservative confidence interval-based analysis, therefore shows that few if any species are significantly associated with any particular week of sampling, and moreover suggests that most species are stochastically distributed through the sampling season.

Table S3.2. Species with abundance significantly associated with particular weeks of the year based on Empirical Bayes approach, for total sample dataset and dataset less species which occur in only one or two sampling events.

| **Week** | | **Total** | | **No Single/Doubleton** | |
| --- | --- | --- | --- | --- | --- |
|  | |  | |  | |
| 19 | *Protomagnathus americanus** | |  | |  |
| **20** | ***Lasius latipes*** | | ***Lasius latipes*** | |  |
| 21 | *Aphaenogaster rudis Complex* | | *Aphaenogaster rudis Complex* | |  |
| 21 | *Lasius umbratus* | |  | |  |
| 21 |  | | *Monomorium emarginatum* | |  |
| 23 | *Formica dolosa* | |  | |  |
| 23 |  | | *Crematogaster lineolata* | |  |
| 27 | *Monomorium emarginatum* | |  | |  |
| 27 |  | | *Myrmica “sculptilis”* | |  |
| 29 | *Myrmica “smithana”* | | *Myrmica “smithana”* | |  |
| **33** | ***Amblyopone pallipes*** | | ***Amblyopone pallipes*** | |  |
| **33** | ***Lasius latipes*** | |  | |  |
| **33** |  | | ***Lasius nearcticus*** | |  |
| **38** | ***Lasius claviger*** | |  | |  |
| 38 |  | | *Stenamma impar* | |  |
| 38 | *Stenamma schmitti* | |  | |  |
| 38 |  | | *Lasius interjectus* | |  |
| 39 | *Prenolepis impairs* | | *Prenolepis imparis* | |  |
| Species | | 11/**3** | | 11/**3** | |

*Notes:* All listed pairs significant under the mean-based approach, bolded species are significant under the more conservative confidence-interval based approach. Species marked * are singletons or doubletons. All p<0.05.


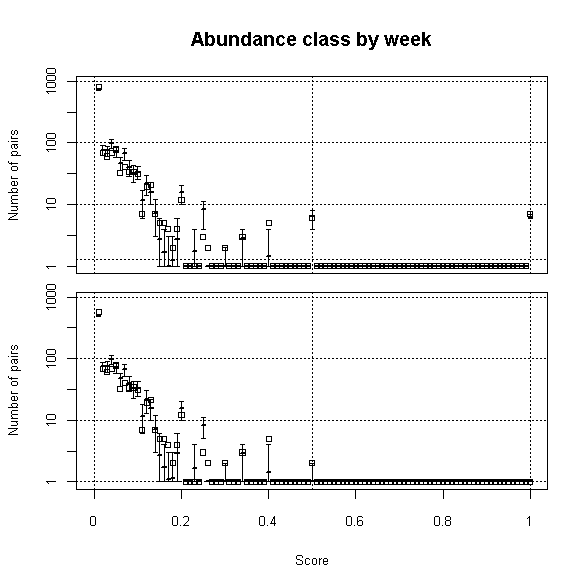


Fig. S3.2. Results from modified empirical Bayes approach for identifying non-random species associations. Lower chart is the same analysis repeated for the dataset with all singletons and doubletons removed. Horizontal axis shows fraction of species’ occurrences in a particular week, whereas vertical axis shows number of pairs in each bin. Open squares show observed values, whereas lines show mean outputs from randomly generated communities (± 1SD). Open squares above the confidence interval are potentially significant species associations.

**REFERENCES IN APPENDIX S3:**

1. Venegas SA, Mysak LA, Straub DN (1996) Evidence for interannual and interdecadal climate variability in the South Atlantic (vol 23, pg 2673, 1996). Geophysical Research Letters 23: 3425-3425.

2. Björnsson H, Venegas SA (2000) A Manual for EOF and SVD analyses of Climatic Data. Department of Atmospheric and Oceanic Sciences and Centre for Climate and Global Change Research McGill University.

3. Gotelli NJ, Ulrich W (2010) The empirical Bayes approach as a tool to identify non-random species associations. Oecologia 162: 463-477.

4. Ellison AM, Record S, Arguello A, Gotelli NJ (2007) Rapid inventory of the ant assemblage in a temperate hardwood forest: species composition and sampling methods. Environ Entomol 36: 766-775.

5. Ellison AM, Farnsworth EJ, Gotelli NJ (2002) Ant diversity in pitcher-plant bogs of Massachusetts. Northeast Nat 9: 267-284.

6. Dunn RR, Parker C, Sanders N (2007) Temporal patterns of diversity: assessing the biotic and abiotic controls on ant assemblages. Biol J Linn Soc 91: 191-201.

7. Gotelli NJ, Ellison AM (2002) Biogeography at a regional scale: Determinants of ant species density in New England bogs and forests. Ecology 83: 1604-1609.

8. Gotelli NJ, Ellison AM (2002) Assembly rules for New England ant assemblages. Oikos 99: 591-599.
