# Supplementary material for: The Effects of Biogeography on Ant Diversity and Activity on the Boston Harbor Islands, Massachusetts, U.S.A
Source: PLoS One. 2011 Nov 29;6(11):e28045. doi: 10.1371/journal.pone.0028045 (PMC3226633; doi:10.1371/journal.pone.0028045)
Supplement: Supporting Information S4 — Effects of Disturbance. (DOCX) [file pone.0028045.s004.docx]

**S4. EFFECTS OF DISTURBANCE**

Many studies suggest that ants are highly sensitive to human disturbance of sites. Areas heavily used or developed by humans tend to have lower species richness, more invasive ants, and to be dominated by so-called “tramp” or “anthrophilic” ants [1-3]. The BHI offer an excellent system in which to estimate the effects of disturbance on ant diversity due to the islands unique land use histories. Four pairs of islands with similar biogeographic properties (i.e. area, isolation from the mainland, and location in Boston Harbor) turn out to be useful “pairs” for this study, in which one has a stronger history of disturbance than the other: Bumpkin and Grape, Calf and Great Brewster, Langlee and Ragged, and Thompson and Spectacle. In all cases, the former is “less disturbed” than the latter.

Both Bumpkin and Grape were used for farming and grazing during the American colonial era, but these practices continued much longer – over 300 years – on Grape Island, which was farmed and grazed into the 1940’s [4]. Calf, though home to a handful of single family homes and shacks (and “illegal boxing matches” in the late 19^th^ century), was left more or less undeveloped, and is currently a major nesting site for shorebirds in the early summer. Great Brewster, on the other hand, was heavily developed in WWII as part of an observation post for US soldiers, the remnants of which still honeycomb the island with tunnels and bunkers [4]. Langlee was left more or less undeveloped since the end of the 19^th^ century and has extensive tree cover, whereas Ragged was used as part of a tourist resort in Hingham Harbor in the late 1800’s, and housed, among other things, a restaurant and “rustic observation shelters” [4]. Finally, while Thompson is currently the site of an Outward Bound recreation camp and includes large mowed game fields, there are still stretches of the island which are forested and undeveloped. Spectacle on the other hand, served as receptacle for the tailings from Boston’s “Big Dig”, and was as a result entirely capped and covered with dirt and clay in the late 1990’s.

In addition to analyzing island pairs, in 2009 we established small experimental plots on six of the islands to measure the effects of disturbance on ant diversity (Calf, Great Brewster, Langlee, Ragged, Thompson and Spectacle). Depending on island size, we selected six to nine one-meter-square plots, and collected all ant species present in the plot using a combination of leaf litter sifting and carbohydrate baiting using Kellogg’s Pecan Sandy cookies. We then cleared all vegetation and removed any ant nests from the plot, leaving a bare, uninhabited patch. Finally, we returned six to eight weeks later to repeat the same sampling procedure, and measure any changes in ant diversity which may have taken place.

Together, these two measurements of disturbance account for long-term land use changes on the scale of islands, and short-term intensive plot-scale disturbances. Though there was a general trend towards lower ant diversity on islands and plots with higher levels of disturbance, the differences were not significant. This was true both for total number of species and mean number of species per sampling event. The single exception is Spectacle Island, which was more or less defaunated following its capping and redevelopment by the NPS, and is therefore understandably significantly less species rich than its island pair, Thompson. Again, however, mean number of species per sampling event was not significantly different. These two studies therefore suggest that except in extreme cases, anthropogenic disturbance as typically seen on the BHI does not lead to changes in ant species richness, except in extreme circumstances, such as the eradication of all species on an island.


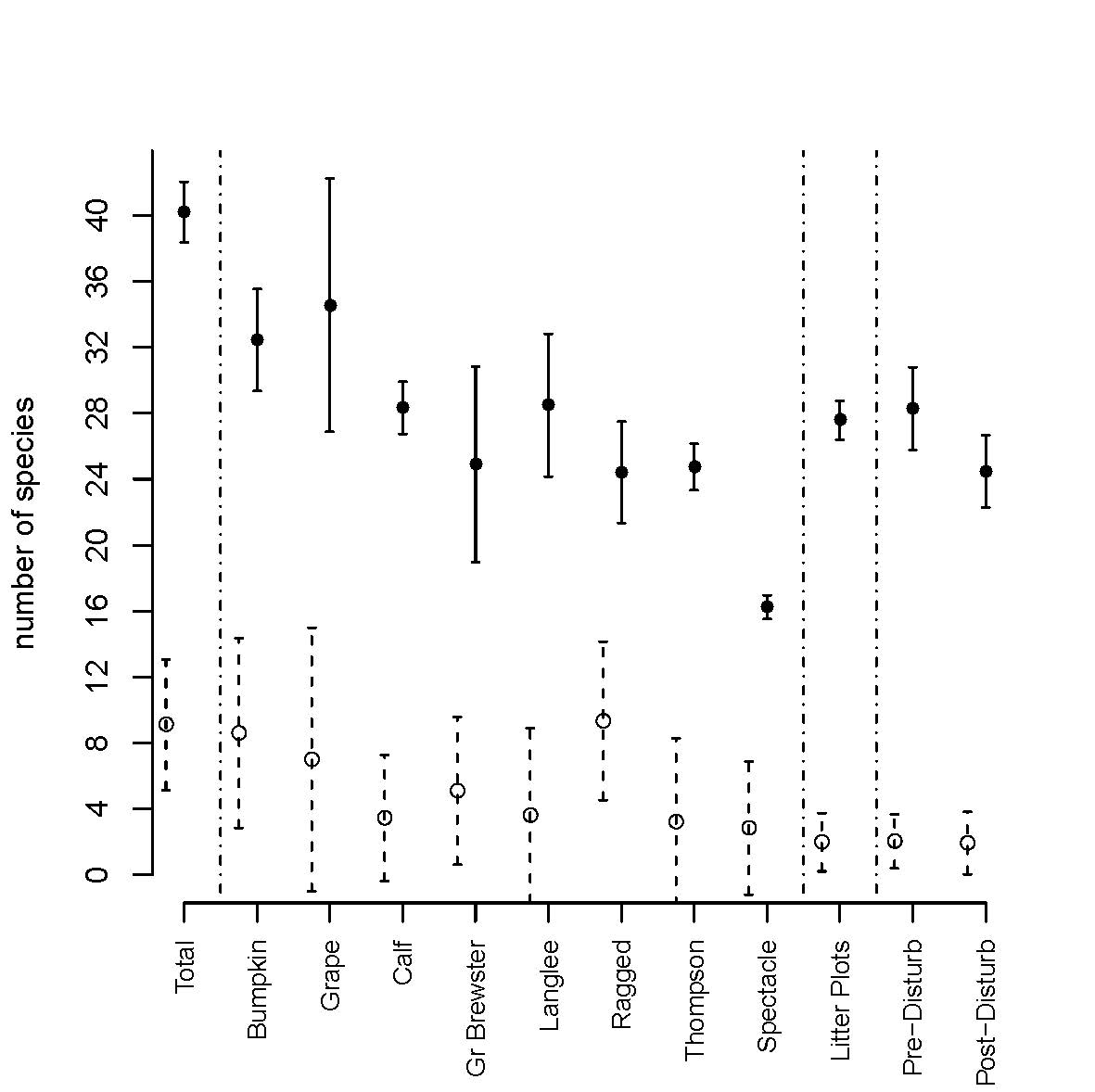


Fig. S4. Diversity metrics for disturbance analysis on islands in the BHI. Closed circles and black lines represent estimated number of active species, open circles and dashed lines show estimated mean species per sampling event. Intervals represent ±1 SD. “Litter plots”, and the pre- and post- disturbance plots summarize the results of a 2009 plot disturbance experiment. See text for further explanation.

**REFERENCES IN APPENDIX S4:**

1. Rizali A, Lohman DJ, Buchori D, Prasetyo LB, Triwidodo H, et al. (2010) Ant communities on small tropical islands: effects of island size and isolation are obscured by habitat disturbance and 'tramp' ant species. J Biogeogr 37: 229-236.

2. Schoereder JH, Sobrinho TG, Ribas CR, Campos RBF (2004) Colonization and extinction of ant communities in a fragmented landscape. Austral Ecol 29: 391-398.

3. Suarez AV, Bolger DT, Case TJ (1998) Effects of fragmentation and invasion on native ant communities in coastal southern California. Ecology 79: 2041-2056.

4. NPS (2011) Island Facts. In: NPS, editor. Boston Harbor Islands History and Culture.
