# Supplementary material for: The Effects of Biogeography on Ant Diversity and Activity on the Boston Harbor Islands, Massachusetts, U.S.A
Source: PLoS One. 2011 Nov 29;6(11):e28045. doi: 10.1371/journal.pone.0028045 (PMC3226633; doi:10.1371/journal.pone.0028045)
Supplement: Supporting Information S5 — BHI Habitat Maps. (DOCX) [file pone.0028045.s005.docx]

**S5. BHI HABITAT MAPS**

Habitat Inventory

To account for differences in diversity due to habitat, we recorded vegetation and habitat type at each sample site. On six of the ten islands, we completed more extensive vegetation surveys and created habitat community maps for the entire ground cover. We obtained habitat community classifications from the National Heritage Program’s Natural Communities Classification [1] and a list of vegetation communities found on each island from Elliman *et al.’s* plant species list for the BHI [2]. We completed on-site ground truthing to mark the borders between habitat community types using a Garmin handheld GPS etrex unit. We later mapped these boundaries with the aid of aerial photos from MassGIS [3] in ArcGIS 9.3 using the ArcSketch package.

We identified 18 distinct plant communities on six islands. General trends ranged from the highly habitat-diverse Thompson Island to the shrubby Spectacle Island to the small yet greatly forested Langlee and Ragged Islands. In order to increase simplify the maps for display purposes, we collapsed the 18 plant community categories into 5 based on sampling frequency and previous understanding that ant communities should differ greatly between these habitat types [4-6]. The full map, with all 18 plant community categories, is deposited with the NPS, and are available at request from the authors.

Fig. S5. Course habitat map of ATBI sites on the BHI.

**REFERENCES IN APPENDIX:**

1. Swain PC, Kearsley JB (2001) Classification of the Natural Communities of Massachusetts. Version 1.3. Natural Heritage & Endangered Species Program, Massachusetts Division of Fisheries & Wildlife. Westborough, MA.

2. Elliman T (2005) Vascular flora and plant communities of the Boston Harbor Islands. Northeast Nat 12: 49-76.

3. MassGIS (2010) Office of Geographic and Environmental Information, Commonwealth of Massachusetts Executive Office of Energy and Environmental Affairs.

4. Gotelli NJ, Ellison AM (2002) Assembly rules for New England ant assemblages. Oikos 99: 591-599.

5. Kaspari M, Agosti D, Majer JD, Alonso LE, Schultz TR (2000) A primer on ant ecology. Ants: standard methods for measuring and monitoring biodiversity: 9-24, 231-269.

6. Wilson EO (1964) The ants of the Florida Keys. Breviora 210: 1-14.
