# Supplementary material for: The Effects of Biogeography on Ant Diversity and Activity on the Boston Harbor Islands, Massachusetts, U.S.A
Source: PLoS One. 2011 Nov 29;6(11):e28045. doi: 10.1371/journal.pone.0028045 (PMC3226633; doi:10.1371/journal.pone.0028045)
Supplement: Supporting Information S6 — ANOVA tables (Worlds End included in analysis). (DOCX) [file pone.0028045.s006.docx]

**Appendix S6** **ANOVA TABLES (Words End included in analysis)**

**1. Weekly estimated number of active species**

a) vs. isolation

Estimate Std. Error t value Pr(>|t|)

(Intercept) 14.567210 2.604184 5.594 1.98e-07 ***

week 0.006848 0.081154 0.084 0.933

isolation 4.319591 1.051220 4.109 8.20e-05 ***

---

Residual standard error: 4.916 on 99 degrees of freedom

Multiple R-squared: 0.1466, Adjusted R-squared: 0.1294

F-statistic: 8.504 on 2 and 99 DF, p-value: 0.0003907

b) vs. size

Estimate Std. Error t value Pr(>|t|)

(Intercept) 19.02449 2.75718 6.900 4.98e-10 ***

week 0.03169 0.08433 0.376 0.708

size -3.15570 1.12369 -2.808 0.006 **

---

Residual standard error: 5.119 on 99 degrees of freedom

Multiple R-squared: 0.07477, Adjusted R-squared: 0.05607

F-statistic: 4 on 2 and 99 DF, p-value: 0.02135

c) vs. isolation and interactions

Estimate Std. Error t value Pr(>|t|)

(Intercept) 12.26715 4.93999 2.483 0.0147 *

week 0.08288 0.16074 0.516 0.6073

isolation 7.43597 5.77704 1.287 0.2011

week:isolation -0.10230 0.18644 -0.549 0.5845

---

Residual standard error: 4.933 on 98 degrees of freedom

Multiple R-squared: 0.1492, Adjusted R-squared: 0.1232

F-statistic: 5.729 on 3 and 98 DF, p-value: 0.001180

d) vs. size and interactions

Estimate Std. Error t value Pr(>|t|)

(Intercept) 16.0393 4.6229 3.470 0.000776 ***

week 0.1290 0.1474 0.875 0.383785

size 1.2987 5.6449 0.230 0.818514

week:size -0.1449 0.1799 -0.805 0.422603

---

Residual standard error: 5.128 on 98 degrees of freedom

Multiple R-squared: 0.08085, Adjusted R-squared: 0.05271

F-statistic: 2.873 on 3 and 98 DF, p-value: 0.04014

**2. Number of species per plot**

a) vs. isolation

Estimate Std. Error t value Pr(>|t|)

(Intercept) 1.857782 0.489172 3.798 0.000252 ***

week -0.005885 0.015235 -0.386 0.700089

isolation 0.609241 0.198367 3.071 0.002752 **

---

Residual standard error: 0.9279 on 99 degrees of freedom

Multiple R-squared: 0.08734, Adjusted R-squared: 0.0689

F-statistic: 4.737 on 2 and 99 DF, p-value: 0.01085

b) vs. size

Estimate Std. Error t value Pr(>|t|)

(Intercept) 2.008809 0.511656 3.926 0.000160 ***

week -0.003826 0.015774 -0.243 0.808841

size 0.288558 0.209154 1.380 0.170806

---

Residual standard error: 0.9619 on 99 degrees of freedom

Multiple R-squared: 0.01924, Adjusted R-squared: -0.0005769

F-statistic: 0.9709 on 2 and 99 DF, p-value: 0.3823

c) vs. isolation and interactions

Estimate Std. Error t value Pr(>|t|)

(Intercept) 1.770777 0.933834 1.896 0.0609 .

week -0.003009 0.030386 -0.099 0.9213

isolation 0.726619 1.089491 0.667 0.5064

week:isolation -0.003855 0.035178 -0.110 0.9130

---

d) vs. size and interactions

Estimate Std. Error t value Pr(>|t|)

(Intercept) 1.17772 0.84395 1.395 0.166

week 0.02345 0.02710 0.865 0.389

size 1.54842 1.04012 1.489 0.140

week:size -0.04115 0.03328 -1.236 0.219

Residual standard error: 0.9594 on 98 degrees of freedom

Multiple R-squared: 0.0343, Adjusted R-squared: 0.004738

F-statistic: 1.16 on 3 and 98 DF, p-value: 0.3289

**3. Spatial turnover (plot dissimilarity)**

a) vs. isolation

Estimate Std. Error t value Pr(>|t|)

(Intercept) 7.71411 1.80835 4.266 4.63e-05 ***

week 0.05937 0.05644 1.052 0.295

isolation -0.59977 0.72448 -0.828 0.410

---

Residual standard error: 3.372 on 97 degrees of freedom

Multiple R-squared: 0.01704, Adjusted R-squared: -0.003223

F-statistic: 0.841 on 2 and 97 DF, p-value: 0.4344

b) vs. size

Estimate Std. Error t value Pr(>|t|)

(Intercept) 9.38005 1.74170 5.386 5.04e-07 ***

week 0.05365 0.05283 1.016 0.312355

sizeS -2.63541 0.70456 -3.740 0.000311 ***

---

Residual standard error: 3.163 on 97 degrees of freedom

Multiple R-squared: 0.1349, Adjusted R-squared: 0.117

F-statistic: 7.562 on 2 and 97 DF, p-value: 0.0008874

c) vs. isolation and interactions

Estimate Std. Error t value Pr(>|t|)

(Intercept) 8.2062 3.3933 2.418 0.0175 *

week 0.0431 0.1104 0.390 0.6971

isolation -1.2734 3.9895 -0.319 0.7503

week:isolation 0.0221 0.1287 0.172 0.8640

---

Residual standard error: 3.389 on 96 degrees of freedom

Multiple R-squared: 0.01735, Adjusted R-squared: -0.01336

F-statistic: 0.5649 on 3 and 96 DF, p-value: 0.6395

d) vs. size and interactions

Estimate Std. Error t value Pr(>|t|)

(Intercept) 9.178000 2.966005 3.094 0.00258 **

week 0.060180 0.093802 0.642 0.52268

size -2.338598 3.587833 -0.652 0.51608

week:size -0.009603 0.113794 -0.084 0.93292

---

Residual standard error: 3.18 on 96 degrees of freedom

Multiple R-squared: 0.1349, Adjusted R-squared: 0.1079

F-statistic: 4.992 on 3 and 96 DF, p-value: 0.002922

**4. Temporal turnover (Bray-Curtis distance)**

a) vs. isolation

Estimate Std. Error t value Pr(>|t|)

(Intercept) 0.6024873 0.0993092 6.067 3.03e-08 ***

week 0.0007118 0.0030484 0.234 0.8159

isolation -0.0875145 0.0365184 -2.396 0.0186 *

---

Residual standard error: 0.1626 on 90 degrees of freedom

(10 observations deleted due to missingness)

Multiple R-squared: 0.06, Adjusted R-squared: 0.03911

F-statistic: 2.872 on 2 and 90 DF, p-value: 0.06176

b) vs. size

Estimate Std. Error t value Pr(>|t|)

(Intercept) 0.5134587 0.1000516 5.132 1.64e-06 ***

week -0.0002632 0.0030497 -0.086 0.9314

size 0.0854169 0.0368933 2.315 0.0229 *

---

Residual standard error: 0.163 on 90 degrees of freedom

(10 observations deleted due to missingness)

Multiple R-squared: 0.05623, Adjusted R-squared: 0.03526

F-statistic: 2.681 on 2 and 90 DF, p-value: 0.07396

c) vs. isolation and interactions

Estimate Std. Error t value Pr(>|t|)

(Intercept) 6.241e-01 1.881e-01 3.317 0.00132 **

week 1.568e-05 5.983e-03 0.003 0.99792

isolation -1.170e-01 2.210e-01 -0.530 0.59773

week:isolation 9.439e-04 6.967e-03 0.135 0.89253

---

Residual standard error: 0.1635 on 89 degrees of freedom

(10 observations deleted due to missingness)

Multiple R-squared: 0.0602, Adjusted R-squared: 0.02852

F-statistic: 1.9 on 3 and 89 DF, p-value: 0.1353

d) vs. size and interactions

Estimate Std. Error t value Pr(>|t|)

(Intercept) 0.326377 0.161424 2.022 0.0462 *

week 0.005730 0.005078 1.129 0.2621

size 0.378220 0.202400 1.869 0.0650 .

week:size -0.009308 0.006328 -1.471 0.1448

---

Residual standard error: 0.1619 on 89 degrees of freedom

(10 observations deleted due to missingness)

Multiple R-squared: 0.07863, Adjusted R-squared: 0.04757

F-statistic: 2.532 on 3 and 89 DF, p-value: 0.06212
