# Supplementary material for: The Effects of Biogeography on Ant Diversity and Activity on the Boston Harbor Islands, Massachusetts, U.S.A
Source: PLoS One. 2011 Nov 29;6(11):e28045. doi: 10.1371/journal.pone.0028045 (PMC3226633; doi:10.1371/journal.pone.0028045)
Supplement: Supporting Information S7 — ANOVA tables (Worlds End NOT included in analysis). (DOCX) [file pone.0028045.s007.docx]

**Appendix S7.** **ANOVA TABLES (Words End NOT included in analysis)**


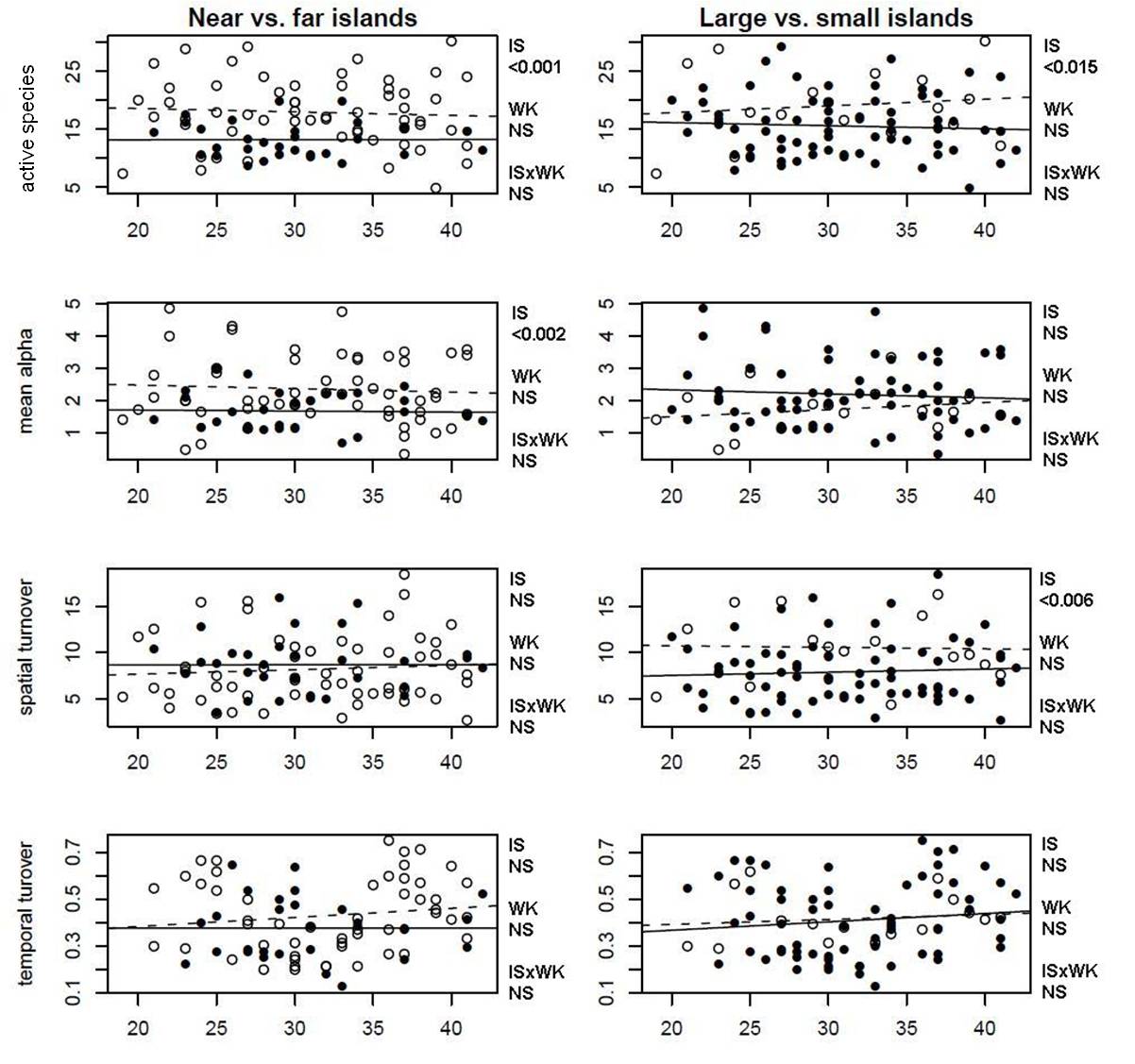


Fig. S7. ANCOVA of island area and isolation per week against estimated number of active species, mean species per sampling event, sampling event heterogeneity, and temporal turnover. “Near” islands are 0-1.65 km and “far” islands are 1.65-3.29 km from the nearest mainland; “small” islands are 0-0.54 km^2^ in terrestrial area above the high tide mark and “large” islands are 0.54-1.08 km^2^. “IS” shows the test statistic for differences between islands, “WK” between weeks, and “ISxWK” the interaction effect. Open circles show near and large islands, closed show small and far.

**1. Weekly estimated number of active species, no Worlds End**

a) vs. isolation

Estimate Std. Error t value Pr(>|t|)

(Intercept) 14.49749 2.82427 5.133 1.72e-06 ***

week -0.04147 0.08860 -0.468 0.641

isolation 4.76068 1.11328 4.276 4.86e-05 ***

---

Residual standard error: 5.039 on 87 degrees of freedom

Multiple R-squared: 0.1738, Adjusted R-squared: 0.1548

F-statistic: 9.151 on 2 and 87 DF, p-value: 0.0002472

b) vs. size

Estimate Std. Error t value Pr(>|t|)

(Intercept) 19.59092 3.18714 6.147 2.32e-08 ***

week -0.01293 0.09388 -0.138 0.8908

size -3.58436 1.44265 -2.485 0.0149 *

---

Residual standard error: 5.357 on 87 degrees of freedom

Multiple R-squared: 0.06639, Adjusted R-squared: 0.04493

F-statistic: 3.093 on 2 and 87 DF, p-value: 0.05037

c) vs. isolation and interactions

Estimate Std. Error t value Pr(>|t|)

(Intercept) 13.160066 5.072247 2.595 0.0111 *

week 0.002744 0.165044 0.017 0.9868

isolation 6.665822 6.091279 1.094 0.2769

week:isolation -0.062374 0.196034 -0.318 0.7511

---

Residual standard error: 5.066 on 86 degrees of freedom

Multiple R-squared: 0.1748, Adjusted R-squared: 0.146

F-statistic: 6.071 on 3 and 86 DF, p-value: 0.0008481

d) vs. size and interactions

Estimate Std. Error t value Pr(>|t|)

(Intercept) 15.5678 6.0928 2.555 0.0124 *

week 0.1168 0.1920 0.609 0.5444

size 1.7054 6.9733 0.245 0.8074

week:size -0.1708 0.2203 -0.775 0.4402

---

Signif. codes: 0 ‘***’ 0.001 ‘**’ 0.01 ‘*’ 0.05 ‘.’ 0.1 ‘ ’ 1

Multiple R-squared: 0.07288, Adjusted R-squared: 0.04053

F-statistic: 2.253 on 3 and 86 DF, p-value: 0.08791

**2. Number of species per plot, no Worlds End**

a) vs. isolation

Estimate Std. Error t value Pr(>|t|)

(Intercept) 1.937111 0.511464 3.787 0.000281 ***

week -0.008508 0.016047 -0.530 0.597360

isolation 0.687039 0.202008 3.401 0.001020 **

---

Residual standard error: 0.9112 on 86 degrees of freedom

Multiple R-squared: 0.1191, Adjusted R-squared: 0.0986

F-statistic: 5.813 on 2 and 86 DF, p-value: 0.004286

b) vs. size

Estimate Std. Error t value Pr(>|t|)

(Intercept) 1.954928 0.572355 3.416 0.000974 ***

week -0.003767 0.016863 -0.223 0.823772

size 0.340602 0.259131 1.314 0.192205

---

Residual standard error: 0.961 on 86 degrees of freedom

Multiple R-squared: 0.02029, Adjusted R-squared: -0.002497

F-statistic: 0.8904 on 2 and 86 DF, p-value: 0.4142

c) vs. isolation and interactions

Estimate Std. Error t value Pr(>|t|)

(Intercept) 1.770777 0.917524 1.930 0.057 .

week -0.003009 0.029855 -0.101 0.920

isolation 0.924445 1.103338 0.838 0.404

week:isolation -0.007768 0.035485 -0.219 0.827

---

Residual standard error: 0.9163 on 85 degrees of freedom

Multiple R-squared: 0.1196, Adjusted R-squared: 0.08851

F-statistic: 3.848 on 3 and 85 DF, p-value: 0.01234

d) vs. size and interactions

Estimate Std. Error t value Pr(>|t|)

(Intercept) 0.59364 1.08308 0.548 0.5851

week 0.04015 0.03413 1.176 0.2428

size 2.13249 1.24051 1.719 0.0892 .

week:size -0.05784 0.03917 -1.477 0.1435

---

Residual standard error: 0.9544 on 85 degrees of freedom

Multiple R-squared: 0.04479, Adjusted R-squared: 0.01108

F-statistic: 1.329 on 3 and 85 DF, p-value: 0.2705

**3. Spatial turnover (plot dissimilarity), no Worlds End**

a) vs. isolation

Estimate Std. Error t value Pr(>|t|)

(Intercept) 7.64845 1.98095 3.861 0.000220 ***

week 0.03387 0.06224 0.544 0.587731

isolation -0.52300 0.77489 -0.675 0.501554

---

Residual standard error: 3.484 on 85 degrees of freedom

Multiple R-squared: 0.008104, Adjusted R-squared: -0.01523

F-statistic: 0.3472 on 2 and 85 DF, p-value: 0.7076

b) vs. size

Estimate Std. Error t value Pr(>|t|)

(Intercept) 9.80913 2.05149 4.781 7.23e-06 ***

week 0.02261 0.05949 0.380 0.70482

size -2.62215 0.92378 -2.838 0.00567 **

---

Residual standard error: 3.339 on 85 degrees of freedom

Multiple R-squared: 0.08913, Adjusted R-squared: 0.0677

F-statistic: 4.159 on 2 and 85 DF, p-value: 0.01892

c) vs. isolation and interactions

Estimate Std. Error t value Pr(>|t|)

(Intercept) 8.624448 3.507180 2.459 0.0160 *

week 0.001606 0.114119 0.014 0.9888

isolation -1.933068 4.243108 -0.456 0.6499

week:isolation 0.046131 0.136456 0.338 0.7362

---

Residual standard error: 3.503 on 84 degrees of freedom

Multiple R-squared: 0.009452, Adjusted R-squared: -0.02592

F-statistic: 0.2672 on 3 and 84 DF, p-value: 0.8489

d) vs. size and interactions

Estimate Std. Error t value Pr(>|t|)

(Intercept) 11.04931 4.04445 2.732 0.00767 **

week -0.01676 0.12560 -0.133 0.89418

size -4.21767 4.57147 -0.923 0.35885

week:size 0.05091 0.14283 0.356 0.72240

---

Residual standard error: 3.356 on 84 degrees of freedom

Multiple R-squared: 0.0905, Adjusted R-squared: 0.05802

F-statistic: 2.786 on 3 and 84 DF, p-value: 0.04569

**4. Temporal turnover (Bray-Curtis distance), no Worlds End**

a) vs. isolation

Estimate Std. Error t value Pr(>|t|)

(Intercept) 0.291956 0.098420 2.966 0.004 **

week 0.002689 0.003041 0.884 0.379

isolation 0.051783 0.035014 1.479 0.143

---

Residual standard error: 0.1501 on 78 degrees of freedom

Multiple R-squared: 0.04064, Adjusted R-squared: 0.01604

F-statistic: 1.652 on 2 and 78 DF, p-value: 0.1983

b) vs. size

Estimate Std. Error t value Pr(>|t|)

(Intercept) 0.314900 0.104452 3.015 0.00347 **

week 0.003200 0.003064 1.044 0.29952

size -0.007434 0.042474 -0.175 0.86152

---

Residual standard error: 0.1522 on 78 degrees of freedom

Multiple R-squared: 0.01413, Adjusted R-squared: -0.01115

F-statistic: 0.5589 on 2 and 78 DF, p-value: 0.5741

c) vs. isolation and interactions

Estimate Std. Error t value Pr(>|t|)

(Intercept) 3.759e-01 1.735e-01 2.167 0.0333 *

week -1.568e-05 5.516e-03 -0.003 0.9977

isolation -7.083e-02 2.112e-01 -0.335 0.7382

week:isolation 3.901e-03 6.624e-03 0.589 0.5576

---

Residual standard error: 0.1508 on 77 degrees of freedom

Multiple R-squared: 0.04494, Adjusted R-squared: 0.007734

F-statistic: 1.208 on 3 and 77 DF, p-value: 0.3126

d) vs. size and interactions

Estimate Std. Error t value Pr(>|t|)

(Intercept) 0.349994 0.197595 1.771 0.0805 .

week 0.002095 0.006106 0.343 0.7325

size -0.054590 0.228863 -0.239 0.8121

week:size 0.001484 0.007073 0.210 0.8344

---

Residual standard error: 0.1531 on 77 degrees of freedom

Multiple R-squared: 0.01469, Adjusted R-squared: -0.0237

F-statistic: 0.3827 on 3 and 77 DF, p-value: 0.7658
