# Supplementary material for: The Effects of Biogeography on Ant Diversity and Activity on the Boston Harbor Islands, Massachusetts, U.S.A
Source: PLoS One. 2011 Nov 29;6(11):e28045. doi: 10.1371/journal.pone.0028045 (PMC3226633; doi:10.1371/journal.pone.0028045)
Supplement: Supporting Information S8 — Boston Harbor Island ant sample data (available online at http://insects.oeb.harvard.edu/boston_islands/). (DOCX) [file pone.0028045.s008.docx]

**Appendix S8.** **BOSTON HARBOR ISLANDS SAMPLE DATA**

available online at http://insects.oeb.harvard.edu/boston_islands/
